# Probing instructions for expression regulation in gene nucleotide compositions

**DOI:** 10.1371/journal.pcbi.1005921

**Published:** 2018-01-02

**Authors:** Chloé Bessière, May Taha, Florent Petitprez, Jimmy Vandel, Jean-Michel Marin, Laurent Bréhélin, Sophie Lèbre, Charles-Henri Lecellier

**Affiliations:** 1 IBC, Univ. Montpellier, CNRS, Montpellier, France; 2 Institut de Génétique Moléculaire de Montpellier, University of Montpellier, CNRS, Montpellier, France; 3 IMAG, Univ. Montpellier, CNRS, Montpellier, France; 4 LIRMM, Univ. Montpellier, CNRS, Montpellier, France; 5 Univ. Paul-Valéry-Montpellier 3, Montpellier, France; University of Toronto, CANADA

## Abstract

Gene expression is orchestrated by distinct regulatory regions to ensure a wide variety of cell types and functions. A challenge is to identify which regulatory regions are active, what are their associated features and how they work together in each cell type. Several approaches have tackled this problem by modeling gene expression based on epigenetic marks, with the ultimate goal of identifying driving regions and associated genomic variations that are clinically relevant in particular in precision medicine. However, these models rely on experimental data, which are limited to specific samples (even often to cell lines) and cannot be generated for all regulators and all patients. In addition, we show here that, although these approaches are accurate in predicting gene expression, inference of TF combinations from this type of models is not straightforward. Furthermore these methods are not designed to capture regulation instructions present at the sequence level, before the binding of regulators or the opening of the chromatin. Here, we probe sequence-level instructions for gene expression and develop a method to explain mRNA levels based solely on nucleotide features. Our method positions nucleotide composition as a critical component of gene expression. Moreover, our approach, able to rank regulatory regions according to their contribution, unveils a strong influence of the gene body sequence, in particular introns. We further provide evidence that the contribution of nucleotide content can be linked to co-regulations associated with genome 3D architecture and to associations of genes within topologically associated domains.

## Author summary

Identifying a maximum of DNA determinants implicated in gene regulation will accelerate genetic analyses and precision medicine approaches by identifying key gene features. In that context decoding the sequence-level instructions for gene regulation is of prime importance. Among global efforts to achieve this objective, we propose a novel approach able to explain gene expression in each patient sample using only DNA features. Our approach, which is as accurate as methods based on epigenetics data, reveals a strong influence of the nucleotide content of gene body sequences, in particular introns. In contrast to canonical regulations mediated by specific DNA motifs, our model unveils a contribution of global nucleotide content notably in co-regulations associated with genome 3D architecture and to associations of genes within topologically associated domains. Overall our study confirms and takes advantage of the existence of sequence-level instructions for gene expression, which lie in genomic regions largely underestimated in regulatory genomics but which appear to be linked to chromatin architecture.

## Introduction

The diversity of cell types and cellular functions is defined by specific patterns of gene expression. The regulation of gene expression involves a plethora of DNA/RNA-binding proteins that bind specific motifs present in various DNA/RNA regulatory regions. At the DNA level, transcription factors (TFs) typically bind 6-8bp-long motifs present in promoter regions, which are close to transcription start site (TSS). TFs can also bind enhancer regions, which are distal to TSSs and often interspersed along considerable physical distance through the genome [1]. The current view is that DNA looping mediated by specific proteins and RNAs places enhancers in close proximity with target gene promoters (for review [2–5]). High-resolution chromatin conformation capture (Hi-C) technology identified contiguous genomic regions with high contact frequencies, referred to as topologically associated domains (TADs) [6]. Within a TAD, enhancers can work with many promoters and, on the other hand, promoters can contact more than one enhancer [5, 7]. Several large-scale data derived from high-throughput experiments (such as ChIP-seq [8], SELEX-seq [9], RNAcompete [10]) can be used to highlight TF/RBP binding preferences and build Position Weight Matrixes (PWMs) [11]. The human genome is thought to encode ∼2,000 TFs [12] and >1,500 RBPs [13]. It follows that gene regulation is achieved primarily by allowing the proper combination to occur i.e. enabling cell- and/or function-specific regulators (TFs or RBPs) to bind the proper sequences in the appropriate regulatory regions. In that context, epigenetics clearly plays a central role as it influences the binding of the regulators and ultimately gene expression [14]. Provided the variety of regulatory mechanisms, deciphering their combination requires mathematical/computational methods able to consider all possible combinations [15]. Several methods have recently been proposed to tackle this problem [16–19]. Although these models appear very efficient in predicting gene expression and identifying key regulators, they mostly rely on experimental data (ChIP-seq, methylation, DNase hypersensitivity), which are limited to specific samples (often to cell lines) and which cannot be generated for all TFs/RBPs and all cell types. These technological features impede from using this type of approaches in a clinical context in particular in precision medicine. In addition, we show here that, although these approaches are accurate, their biological interpretation can be misleading. Finally these methods are not designed to capture regulation instructions that may lie at the sequence-level before the binding of regulators or the opening of the chromatin. There is indeed a growing body of evidence suggesting that the DNA sequence *per se* contains information able to shape the epigenome and explain gene expression [20–25]. Several studies have shown that sequence variations affect histone modifications [21–23]. Specific DNA motifs can be associated with specific epigenetic marks and the presence of these motifs can predict the epigenome in a given cell type [24]. Quante and Bird proposed that proteins able to “read” domains of relatively uniform DNA base composition may modulate the epigenome and ultimately gene expression [20]. In that view, modeling gene expression using only DNA sequences and a set of predefined DNA/RNA features (without considering experimental data others than expression data) would be feasible. In line with this proposal, Raghava and Han developed a Support Vector Machine (SVM)-based method to predict gene expression from amino acid and dipeptide composition in *Saccharomyces cerevisiae* [26].

Here, we built a global regression model per sample to explain the expression of the different genes using their nucleotide compositions as predictive variables. The idea beyond our approach is that the selected variables (defining the model) are specific to each sample. Hence the expression of a given gene may be predicted by different variables in different samples. This approach was tested on several independent datasets: 2,053 samples from The Cancer Genome Atlas (1,512 RNA-sequencing data and 582 microarrays) and 3 ENCODE cell lines (RNA sequencing). When restricted to DNA features of promoter regions our model showed accuracy similar to that of two independent methods based on experimental data [17, 19]. We confirmed the importance of nucleotide composition in predicting gene expression. Moreover the performance of our approach increases by combining the contribution of different types of regulatory regions. We thus showed that the gene body (introns, CDS and UTRs), as opposed to sequences located upstream (promoter) or downstream, had the most significant contribution in our model. We further provided evidence that the contribution of nucleotide composition in predicting gene expression is linked to co-regulations associated with genome architecture and TADs.

## Materials and methods

### Datasets, sequences and online resources

RNA-seq V2 level 3 processed data were downloaded from the TCGA Data Portal. Our training data set contained 241 samples randomly chosen from 12 different cancers (20 cancerous samples for each cancer except 21 for LAML). Our model was further evaluated on an additional set of 1,270 tumors from 14 cancer types. We also tested our model on 582 TCGA microarray data. The TCGA barcodes of the samples used in our study have been made available at http://www.univ-montp3.fr/miap/~lebre/IBCRegulatoryGenomics.

Isoform expression data (.rsem.isoforms.normalized_results files) were downloaded from the Broad TCGA GDAC (http://gdac.broadinstitute.org) using firehose_get. We collected data for 73599 isoforms in 225 samples of the 241 initially considered. All the genes and isoforms not detected (no read) in any of the considered samples were removed from the analyses. Expression data were log transformed.

All sequences were mapped to the hg38 human genome and the UCSC liftover tool was used when necessary. Gene TSS positions were extracted from GENCODEv24. UTR and CDS coordinates were extracted from ENSEMBL Biomart. To assign only one 5UTR sequence to one gene, we merged all annotated 5UTRs associated with the gene of interest using Bedtools merge [27] and further concatenated all sequences. The same procedure was used for 3UTRs and CDSs. Intron sequences are GENCODEv24 genes to which 5UTR, 3UTR and CDS sequences described above were substracted using Bedtools substract [27]. These sequences therefore corresponded to constitutive introns. The intron sequences were concatenated per gene. The downstream flanking region (DFR) was defined as the region spanning 1kb after GENCODE v24 gene end. Fasta files were generated using UCSC Table Browser or Bedtools getfasta [27].

TCGA isoform TSSs were retrieved from https://webshare.bioinf.unc.edu/public/mRNAseq_TCGA/unc_hg19.bed and converted into hg38 coordinates with UCSC liftover. For other regulatory regions associated to transcript isoforms (UTRs, CDS, introns and DFR), we used GENCODE v24 annotations.

### Nucleotide composition

The nucleotide (n = 4) and dinucleotide (n = 16) percentages were computed from the different regulatory sequences where:

$$\begin{array}{c} percentage\left(N,s\right)=\frac{♯N}{l} \end{array}$$

is the percentage of nucleotide *N* in the regulatory sequence *s*, with *N* in {*A*, *C*, *G*, *T*} and *l* the length of sequence *s*, and

$$\begin{array}{c} percentage\left(NpM,s\right)=\frac{♯NpM}{l-1} \end{array}$$

is the *NpM* dinucleotide percentage in the regulatory sequence *s*, with *N* and *M* in {*A*, *C*, *G*, *T*} and *l* the length of sequence *s*.

### Motif scores

Motif scores in core promoters were computed using the method explained in [11] and Position Weight Matrix (PWM) available in JASPAR CORE 2016 database [28]. Let *w* be a motif and *s* a nucleic acid sequence. For all nucleotide *N* in {*A*, *C*, *G*, *T*}, we denoted by *P*(*N*|*w*_*j*_) the probability of nucleotide *N* in position *j* of motif *w* obtained from the PWM, and by *P*(*N*) the prior probability of nucleotide *N* in all sequences.

The score of motif *w* at position *i* of sequence *s* is computed as follows:

$$\begin{array}{c} score\left(w,s,i\right)=\sum_{j=0}^{|w|-1}\log\frac{P(s_{i+j}|w_{j})}{P(s_{i+j})} \end{array}$$

with |*w*| the length of motif *w*, *s*_*i*+*j*_ the nucleotide at position *i* + *j* in sequence *s*, The score of motif *w* for sequence *s* is computed as the maximal score that can be achieved at any position of *s*, i.e.:

$$\begin{array}{c} score\left(w,s\right)=\max_{i=0}^{l-|w|}score\left(w,s,i\right), \end{array}$$

with *l* the length of sequence *s*.

Models were also built on sum scores as:

$$\begin{array}{c} scoreSum\left(w,s\right)=\sum_{i=0}^{l-|w|}score\left(w,s,i\right), \end{array}$$

and further compared to models built on mean scores (S1 Fig). Taking mean or sum scores per region yielded similar results (Wilcoxon test p-value = 0.68).

### DNAshape scores

**Fig 1:**
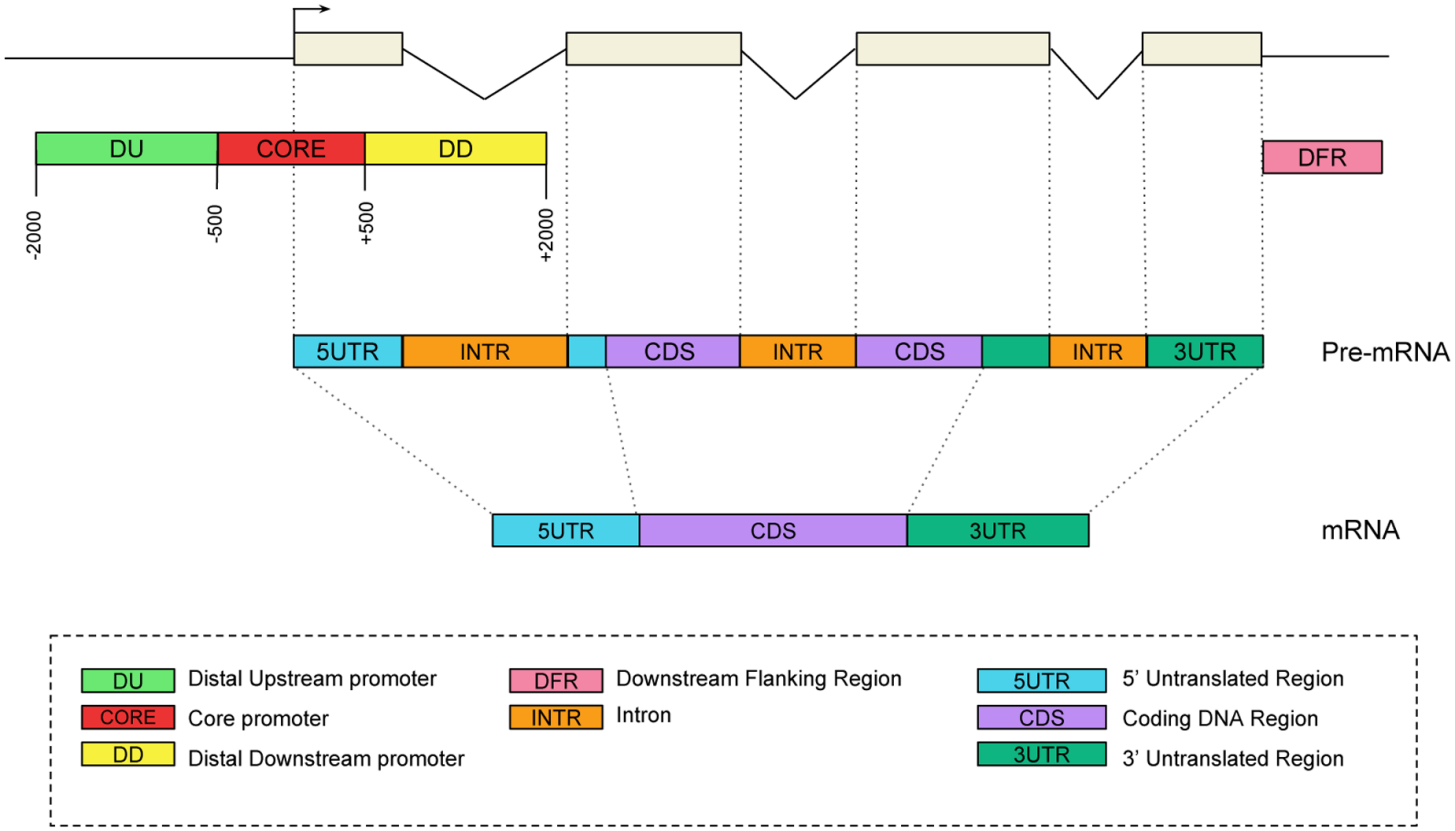
Genomic regions considered for gene expression prediction. An illustrative transcript is shown as example.

DNA shape scores were computed using DNAshapeR [29]. Briefly, provided nucleotide sequences, DNAshapeR uses a sliding pentamer window to derive the structural features corresponding to minor groove width (MGW), helix twist (HelT), propeller twist (ProT) and Roll from all-atom Monte Carlo simulations [29]. Thus, for each DNA shape, a score is given to each base of each sequence considered (DU, CORE and DD—see Fig 1). We then computed the mean of these scores for each sequence providing 12 additional variables per gene.

### Enhancers

The coordinates of the enhancers mapped by FANTOM on the hg19 assembly [7] were converted into hg38 using UCSC liftover and further intersected with the different regulatory regions. We computed the density of enhancers per regulatory region (*R*) by dividing the sum, for all genes, of the intersection length of enhancers with gene $i(L_{enh_{i}})$ by the sum of the lengths of this regulatory region for all genes:

$$\begin{array}{c} enhDensity_{\left(R\right)}=\frac{\sum_{i}\;(L_{enh_{i}}\;\text{in}\;R_{i})}{\sum_{i}\;length\left(R_{i}\right)} \end{array}$$

### Copy Number Variation (CNV)

Processed data were downloaded from the firehose Broad GDAC (https://gdac.broadinstitute.org/). We used the genome-wide SNP array data and the segment mean scores. In order to assign a CNV score to each gene, the coordinates (hg19) of the probes were intersected with that of GENCODE v19 genes using Bedtools intersect [27] and an overlap of 85% of the gene total length. The corresponding segment mean value was then assigned to the intersecting genes. In case no intersection was detected, the gene was assigned a score of 0. We next computed Spearman correlations between genes absolute error (lasso model) and genes absolute segment mean score for each of the 241 samples of the training set.

### Expression quantitative trait loci and single nucleotide polymorphisms

The v6p GTex *cis*-eQTLs were downloaded from the GTex Portal (http://www.gtexportal.org/home/). The hg19 *cis*-eQTL coordinates were converted into hg38 using UCSC liftover and further intersected with the different regulatory regions. We restricted our analyses to *cis*-eQTLs impacting their own host gene. We computed the density of *cis*-eQTL per regulatory region (*R*) by dividing the sum, for all genes, of the number of *cis*-eQTLs of gene *i* (*eQTLs*_*i*_) located in the considered region for gene *i* (*R*_*i*_) by the sum of the lengths of this regulatory region for all genes:

$$\begin{array}{c} eQTLdensity_{\left(R\right)}=\frac{\sum_{i}\;\#(eQTLs_{i}\;\text{in}\;R_{i})}{\sum_{i}\;length\left(R_{i}\right)} \end{array}$$

Likewise we computed the density of SNPs in core promoters and introns by intersecting coordinates of these two regions (liftovered to hg19) with that of SNPs detected on chromosomes 1, 2 and 19 (ftp://ftp.ncbi.nih.gov/snp/organisms/human_9606_b150_GRCh37p13/BED/):

$$\begin{array}{c} SNPdensity_{\left(R\right)}=\frac{\sum_{i}\;\#(SNP_{i}\;\text{in}\;R_{i})}{\sum_{i}\;length\left(R_{i}\right)} \end{array}$$

### Methylation

Illumina Infinium Human DNA Methylation 450 level 3 data were downloaded from the Broad TCGA GDAC (http://gdac.broadinstitute.org) using firehose_get. The coordinates of the methylation sites (hg18) were converted into hg38 using the UCSC liftover and further intersected with that of the core promoters (hg38). For each gene, we computed the median of the beta values of the methylation sites present in the core promoter and further calculated the median of these values in 21 LAML and 17 READ samples with both RNA-seq and methylation data. We compared the overall methylation status of the core promoters in LAML and READ using a wilcoxon test.

### Gini coefficient

We used 8,556 GTEx RNA-seq libraries (https://www.gtexportal.org/home/datasets) to compute the Gini coefficient for 16,134 genes on the 16,294 considered in our model. Gini coefficient measures statistical dispersion and can be used to measure gene ubiquity: value 0 represents genes expressed in all sam- ples while value 1 represents genes expressed in only one sample. To compute Gini coefficient we used R package ineq. We then computed, for the 241 samples, Spearman correlation between Gini coefficients and model gene absolute errors. Similar analyses were performed with 1,897 FANTOM 5 CAGE libraries to compute the Gini coefficients for 15,904 genes.

### Functional enrichment

Gene functional enrichments were computed using the database for annotation, visualization and integrated discovery (DAVID) [30].

### Linear regression with *ℓ*_1_-norm penalty (Lasso)

We performed estimation of the linear regression model (1) via the lasso [31]. Given a linear regression with standardized predictors and centered response values, the lasso solves the *ℓ*_1_-penalized regression problem of finding the vector coefficient *β* = {*β*_*i*_} in order to minimize

$$\begin{array}{c} Min(\left|\right|y^{c}\left(g\right)-\sum_{i}\beta_{i}x_{i,g}^{s}{\left|\right|}^{2}+\lambda \sum_{i}\left|\beta_{i}\right|), \end{array}$$

where *y*^*c*^(*g*) is the centered gene expression for all gene *g*, $x_{i,g}^{s}$ is the standardized DNA feature *i* for gene *g* and ∑_*i*_ |*β*_*i*_| is the *ℓ*_1_-norm of the vector coefficient *β*. Parameter λ is the tuning parameter chosen by 10 fold cross validation. The higher the value of λ, the fewer the variables. This is equivalent to minimizing the sum of squares with a constraint of the form ∑_*i*_ |*β*_*i*_| ≤ *s*. Gene expression predictions are computed using coefficient *β* estimated with the value of λ that minimizes the mean square error. Lasso inference was performed using the function cv.glmnet from the R package glmnet [32]. The LASSO model was compared to two non parametric approaches: Regression trees (CART) [33] and Random forest [34]. S1 Table summarizes accuracy and computing time of each approach. Regression trees achieved significantly lower accuracy than the two other approaches (Wilcox test p-values < 2*e*^−16^), while linear model and random forest yielded similar results (p-value 0.18). Moreover, computing time for linear model was much lower than that of random forest. These results emphasize the merits of linear model such as LASSO in their interpretability and efficiency.

### Variable stability selection

We used the stability selection method developed by Meinshausen *et al.* [35], which is a classical selection method combined with lasso penalization. Consistently selected variables were identified as follows for each sample. First, the lasso inference is repeated 500 times where, for each iteration, (i) only 50% of the genes is used (uniformly sampled) and (ii) a random weight (uniformly sampled in [0.5;1]) is attributed to each predictive variable. Second, a variable is considered as stable if selected in more than 70% of the iterations, using the method proposed in [36] to set the value of lasso penalty λ. One of the advantage of this method is that the variable selection frequency is computed globally for all the variables by attributing a random weight to each variable at each iteration, thus taking into account the dependencies between the variables. This variable stability selection procedure was implemented using functions stabpath and stabsel from the R package C060 for glmnet models [36].

### Regression trees

Regression trees were implemented with the rpart package in R [32]. In order to avoid over-fitting, trees were pruned based on a criterion chosen by cross validation to minimize mean square error. The minimum number of genes was set to 100 genes per leaf.

### TAD enrichment

We considered TADs mapped in IMR90 cells [6] containing more than 10 genes (373 out of 2243 TADs with average number of genes = 14). The largest TAD had 76 associated genes. First, for each TAD and for each region considered, the percentage of each nucleotide and dinucleotide associated to the embedded genes were compared to that of all other genes using a Kolmogorov-Smirnov (KS) test. For a given dinucleotide (for example CpG), we applied KS tests to assess whether the CpG frequency distribution in genes in one specific TAD differs from the distribution in genes in other TADs. Correction for multiple tests was applied using the False Discovery Rate (FDR) < 0.05 [37] and the R function p.adjust [32]. Second, for each of the 967 groups of genes (identified by the regression trees, with mean error < mean error of the 1st quartile), the over-representation of each TAD within each group was tested using the R hypergeometric test function phyper [32]. Correction for multiple tests was applied using FDR< 0.05 [37].

### Availability of data and materials

The matrices of predicted variables (log transformed RNA seq data) and predictive variables (nucleotide and dinucleotide percentages, motifs and DNA shape scores computed for all genes as described above) as well as the TCGA barcodes of the 241 samples used in our study have been made available at http://www.univ-montp3.fr/miap/~lebre/IBCRegulatoryGenomics.

## Results

### Mathematical approach to model gene expression

We built a global linear regression model to explain the expression of genes using DNA/RNA features associated with their regulatory regions (e.g. nucleotide composition, TF motifs, DNA shapes):

$$\begin{array}{c} y\left(g\right)=a+\sum_{i}\;b_{i}x_{i,g}+e\left(g\right) \end{array}$$ (1)

where *y*(*g*) is the expression of gene *g*, *x*_*i*,*g*_ is feature *i* for gene *g*, *e*(*g*) is the residual error associated with gene *g*, *a* is the intercept and *b*_*i*_ is the regression coefficient associated with feature *i*.

The advantage of this approach is that it allows to unveil, into a single model, the most important regulatory features responsible for the observed gene expression. The relative contribution of each feature can thus be easily assessed. It is important to note that the model is specific to each sample. Hence the expression of a given gene may be predicted by different variables depending on the sample. Our computational approach was based on two steps. First, a linear regression model (1) was trained with a lasso penalty [31] to select sequence features relevant for predicting gene expression. Second, the performances of our model was evaluated by computing the mean square of the residual errors, and the correlation between the predicted and the observed expression for all genes. This was done in a 10 fold cross-validation procedure. Namely, in all experiments hereafter, the set of genes was randomly split in ten parts. Each part was alternatively used for the test (i.e. for comparing observed and predicted values) while the remaining genes were used to train the model. This ensures that the model used to predict the expression of a gene has not been trained with any information relative to this gene. Our approach was applied to a set of RNA sequencing data from TCGA. We randomly selected 241 gene expression data from 12 cancer types (see http://www.univ-montp3.fr/miap/~lebre/IBCRegulatoryGenomics for the barcode list). For each dataset (i.e sample), a regression model was learned and evaluated. See Materials and methods for a complete description of the data, the construction of the predictor variables and the inference procedure. We further evaluated our model on 3 independent ENCODE RNA-seq, 1,270 TCGA RNA-seq and 582 microarrays datasets (see below).

### Contribution of the promoter nucleotide composition

**Fig 2:**
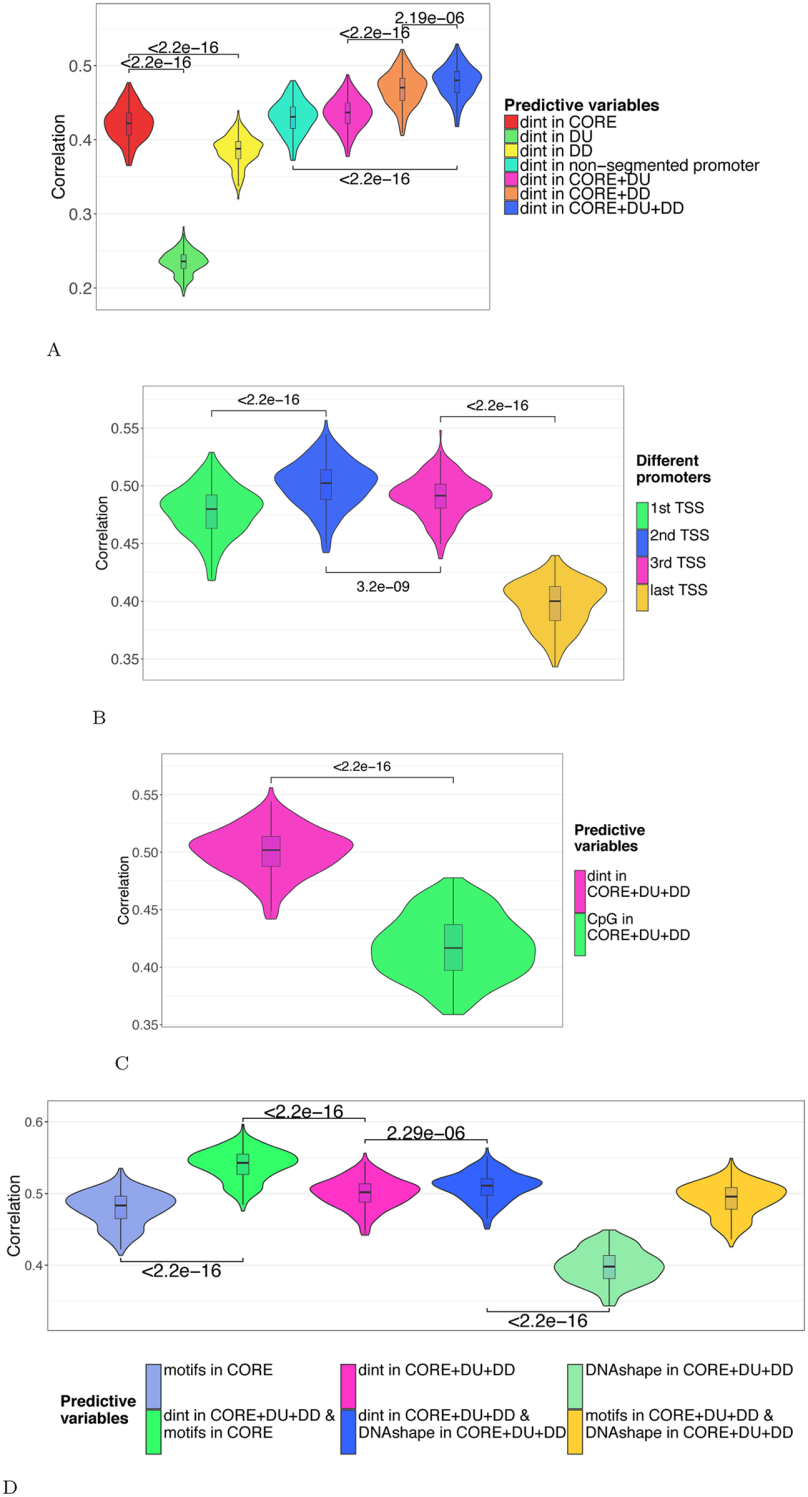
**A: Contribution of the promoter segments.** The model was built using 20 variables corresponding to the nucleotide (4) and dinucleotide (16) percentages computed in the CORE promoter (red), DU (green) or DD (yellow). These variables were then added in different combinations: CORE+DU (pink, 40 variables); CORE+DD (orange, 40 variables); CORE+DU+DD (light blue, 60 variables). Promoter segments were centered around the first most upstream TSS. For sake of comparison, the model was also built on 20 variables corresponding to the nucleotide and dinucleotide compositions of the non segmented promoters (-2000/+2000 around the first most upstream TSS)(light blue). All different models were fitted on 19,393 genes for each of the 241 samples considered. The prediction accuracy was evaluated in each sample by evaluating the Spearman correlation coefficients between observed and predicted gene expressions in a cross-validation procedure. The correlations obtained in all samples are shown as violin plots. **B: Prediction accuracy comparing alternative TSSs.** The model was built using the 60 nucleotide/dinucleotide percentages computed in the 3 promoter segments (CORE+DU+DD) centered around 1st, 2nd, 3rd and last TSSs (from left to right). **C: Contribution of CpG.** The model was built using the 60 nucleotide/dinucleotide or only the 3 CpG percentages computed in the 3 promoter segments (CORE+DU+DD) centered around the 2nd TSS. **D: Contribution of motifs and local DNA shapes.** The model was built using (i) 60 nucleotide/dinucleotide percentages computed in the 3 promoter segments (CORE+DU+DD) (“dint”, pink),(ii) 471 JASPAR2016 PWM scores computed in the CORE segment (“motifs”, light blue) and (iii) the 12 DNA shapes corresponding to the 4 known DNAshapes computed in CORE, DU and DD (“DNAshape”, green). All sequences were centered around the 2nd TSS. These variables were further added in different combinations to build the models indicated: dint+motifs (531 variables, green), dint+DNAshapes (32 variables, dark blue), motifs+DNAshapes (483 variables, light green).

We first evaluated the contribution of promoters, which are one of the most important regulatory sequences implicated in gene regulation [38]. We extracted DNA sequences encompassing ±2000 bases around all GENCODE v24 TSSs and looked at the percentage of dinucleotides along the sequences (S2 Fig). Based on these distributions, we segmented the promoter into three distinct regions: -2000/-500 (referred here to as distal upstream promoter, DU), -500/+500 (thereafter called core promoter though longer than the core promoter traditionally considered) and +500/+2000 (distal downstream promoter, DD)(Fig 1). We computed the nucleotide (n = 4) and dinucleotide (n = 16) relative frequencies in the three distinct regions of each gene. For each sample, we trained one model using the 20 nucleotide/dinucleotide relative frequencies from each promoter segment separately, and from each combination of promoter segments. We observed that the core promoter had the strongest contribution compared to DU and DD (Fig 2A). Considering promoter as one unique sequence spanning -2000/+2000 around TSS achieved lower model accuracy than combining different promoter segments (Fig 2A). The highest accuracy was obtained combining all three promoter segments (Fig 2A).

Promoters are often centered around the 5’ most upstream TSS (i.e. gene start). However genes can have multiple transcriptional start sites. The median number of alternative TSSs for the 19,393 genes listed in the TCGA RNA-seq V2 data is 5 and only 2,753 genes harbor a single TSS (S3 Fig). We therefore evaluated the performance of our model comparing different promoters centered around the first, second, third and last TSS (Fig 2B). In the absence of second TSS, we used the first TSS and likewise the second TSS in the absence of a third TSS. The last TSS represents the most downstream TSS in all cases. We found that our model achieved higher predictive accuracy with the promoters centered around the second TSS (Fig 2B), in agreement with [16]. As postulated by Cheng *et al.* [16] in the case of TFs, the nucleotide composition around the first TSS may be linked to the recruitment of chromatin remodelers and thereby prime the second TSS for gene expression. Dedicated experiments would be required to assess this point.

We noticed that incorporating the number of TSSs associated with each gene drastically increased the performance of our model (S4 Fig). Multiplying TSSs may represent a genuine mechanism to control gene expression level. On the other hand this effect may merely be due to the fact that the more a gene is expressed, the more its different isoforms will be detected (and hence more TSSs will be annotated). Because the number of known TSSs results from annotations deduced from experiments, we decided not to include this variable into our final model.

### Contribution of specific features associated with promoters

Provided the importance of CpGs in promoter activity [38], we first compared our model with a model built only on promoter CpG content. We confirmed that CpG content had an important contribution in predicting gene expression (median R = 0.417, Fig 2C). However considering other dinucleotides achieved better model performances, indicating that dinucleotides other than CpG contribute to gene regulation. This is in agreement with results obtained by Nguyen *et al.*, who showed that CpG content is insufficient to encode promoter activity and that other features might be involved [39].

We integrated TF motifs considering Position Weight Matrix scores computed in the core promoter and observed a slight but significant increase of the regression performance (median r = 0.543 with motif scores vs. r = 0.502 without motif scores, Fig 2D). As DNA sequence is intrinsically linked to three-dimensional local structure of the DNA (DNA shape), we also computed, for each promoter segment (DU, CORE and DD), the mean scores of the four DNA shape features provided by DNAshapeR [29] (helix twist, minor groove width, propeller twist, and Roll), adding 12 variables to the model. Although the difference between models with and without DNA shapes is also significant, the increase in performance is more modest than when including TF motif scores (Fig 2D).

Our model suggested that nucleotide composition had a greater contribution in predicting gene expression compared to TF motifs and DNA shapes. This is in agreement with the findings revealing the influence of the nucleotide environment in TFBS recognition [40]. Note however that nucleotide composition, TF motifs and DNA shapes may be redundant variables. Besides, a linear model may not be optimal to efficiently capture the contributions of TF motifs and/or DNA shapes. The highest performance was achieved by combining nucleotide composition with TF motifs (Fig 2D). In the following analyses, the model was built on both dinucleotide composition and core promoter TF motifs.

### Comparison with models based on experimental data

The wealth of TF ChIP-seq, epigenetic and expression data has allowed the development of methods aimed at predicting gene expression based on differential binding of TFs and epigenetic marks [16–19]. We sought to compare our approach, which does not necessitate such cell-specific experimental data, to these methods. We first compared our results to that of Li *et al.* who used a regression approach called RACER to predict gene expression on the basis of experimental data, in particular TF ChIP-seq data and DNA methylation [17]. Note that, with this model, the contribution of TF regulation in predicting gene expression is higher than that of DNA methylation [17].

**Fig 3:**
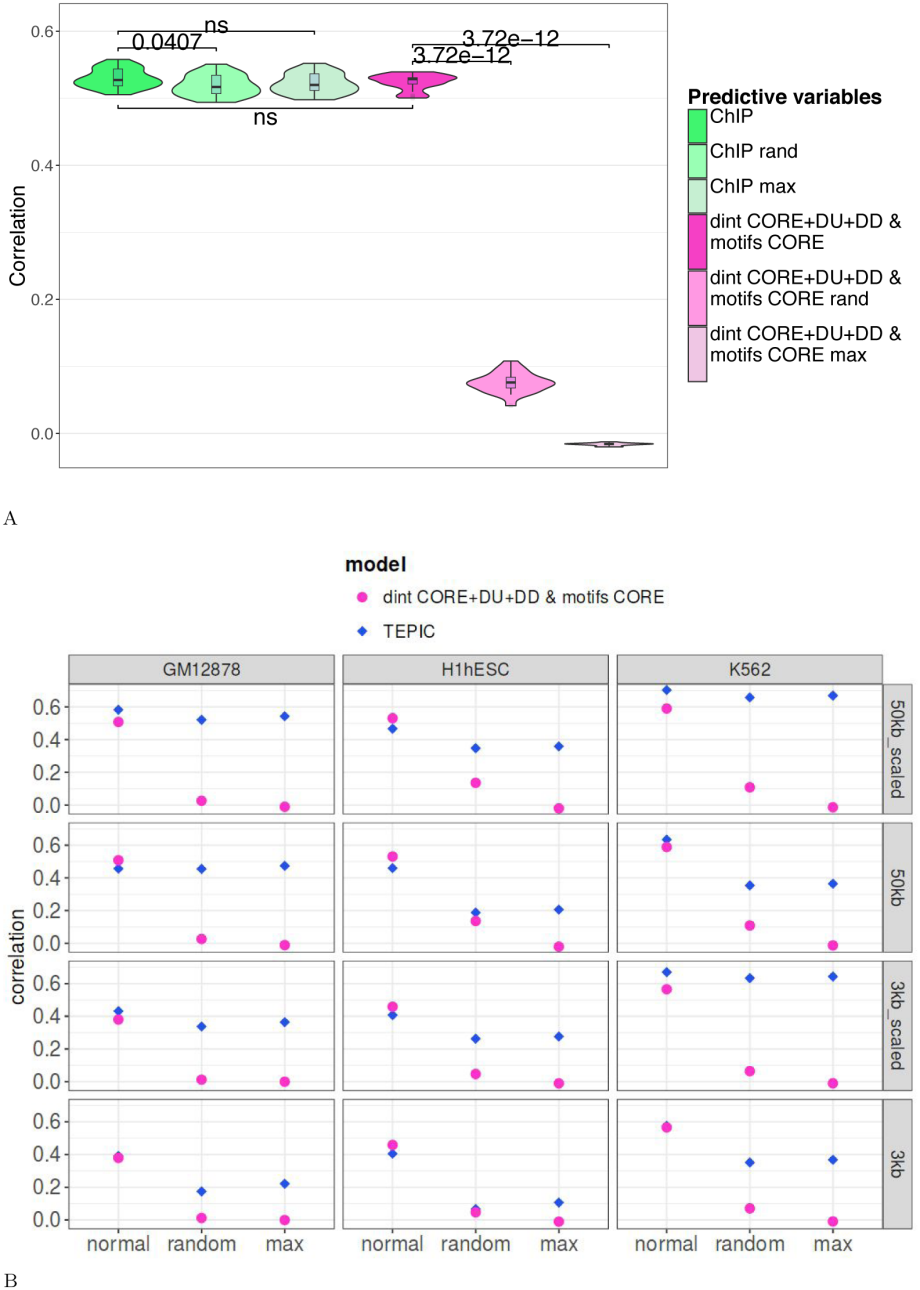
**A: Comparison with model integrating TF-binding signals.** The model was built using 531 variables corresponding to the 60 nucleotide/dinucleotide percentages and the 471 motif scores computed in the 3 promoter segments (CORE, DU, DD) centered around the 2nd TSS (pink). A model built on ChIP-seq data [17] was used for comparison (green). Both models were fitted on the same gene set (n = 16,298) for 21 LAML samples and assessed by cross-validation. The correlations obtained with ChIP-based RACER and our model were compared using Wilcoxon test but no significant difference was observed (p-value = 0.425). The two models were also built on randomized values of predictive variables (rand) and on the maximum value of all predictive variables (max). **B: Comparison with model integrating open-chromatin signals.** The linear model was built using the 531 variables (nucleotide/dinucleotide percentages and motif scores in CORE, DU and DD) and the expression data obtained in K562, hESC and GM12878 [19]. TEPIC was built as described in [19], within a 3 kb or a 50 kb window around TSSs. The scaled version of TEPIC incorporates the abundance of open-chromatin peaks in the analyzed sequences. All types of TEPIC models were tested (3kb, 3kb-scaled, 50kb and 50kb-scaled) by cross-validation. In each case, our model was built on the set of genes considered by TEPIC. TEPIC uses 12 conditions making hard to compute Wilcoxon tests. A direct comparison showed that, in “normal” conditions (first column of each panel), our model and TEPIC give overall very similar results (our model being as accurate as TEPIC in 2 conditions and slightly better in 5 out of the 10 remaining conditions). Models were further built on randomized values of predictive variables (rand) and on the maximum value of all predictive variables (max). Overall, absence of effect of the randomization procedure suggests that RACER and TEPIC mainly capture the level of chromatin opening rather than the TF combinations responsible for gene expression.

We computed the Spearman correlations between expressions observed in the subsets of LAMLs studied in [17] and expressions predicted by our model or by RACER (Fig 3A). For the sake of comparison, we used the RACER model built solely on ChIP-seq data, hereafter referred to as “ChIP-based model”. RACER performance was assessed using the same cross- validation procedure we used for our method. Overall our model was as accurate as ChIP-based model (median correlation r = 0.529 with our model vs. median r = 0.527 with ChIP-based model (Fig 3A)). We then controlled the biological information retrieved by the two approaches by randomly permuting, for each gene, the values of the predictive variables (dinucleotide counts/motif scores in our model and ChIP-seq signals in the ChIP-based model). This creates a situation where the links between the combination of predictive variables and expression is broken, while preserving the score distribution of the variables associated with each gene. For example, genes associated with numerous ChIP-seq peaks will also have numerous ChIP-seq peaks in random data. In such situation, a regression model is expected to poorly perform. Surprisingly, the accuracy of ChIP-based model was not affected by the randomization process (median r = 0.517, Fig 3A) while that of our model was severely impaired (median r = 0.076, Fig 3A). We built another control model using a single predictive variable per gene corresponding to the maximum value of all predictive variables initially considered. Here again the ChIP-based model was not affected by this process (median r = 0.520, Fig 3A) while our model failed to accurately predict gene expression with this type of control variable (median r = -0.016, Fig 3A).

ChIP-seq data are probably the best way to measure the activity of a TF because binding of DNA reflects the output of RNA/protein expression as well as any appropriate post-translational modifications and subcellular localizations. However this type of data also reflects chromatin accessibility (i.e. most TFs bind accessible genomic regions) and TFs tend to form clusters on regulatory regions [41]. The binding of one TF in the promoter region is therefore likely accompanied by the binding of others. Hence, rather than inferring the TF combination responsible for gene expression, linear models based of ChIP-seq data predominantly captures the quantity of TFs (i.e. the opening of the chromatin) in the promoter region of each gene, which explains their good accuracy on randomized or maximized variables.

We indeed observed a similar bias in the results obtained by TEPIC [19], a regression method that predicts gene expression from PWM scores and open-chromatin data. Specifically, TEPIC computes a TF-affinity score for each gene and each PWM by summing up the TF affinities in all open-chromatin peaks (DNaseI-seq) within a close (3,000 bp) or large (50,000 bp) window around TSSs. This scoring takes into account the scores of PWMs in the open-chromatin peaks but is also influenced by the number of open-chromatin peaks in the analyzed sequences and the abundance of open-chromatin peaks (“scaled” version). As a result, genes with many open-chromatin peaks tend to get higher TF-affinity scores than genes with low number of open-chromatin peaks. We trained linear models on three cell-lines using either the four TEPIC affinity-scores or our variables and compared the results (Fig 3B). As for the ChIP-based models, we observed that our model was approximately as accurate as TEPIC score model, validating our approach with an independent dataset. Applying the random permutations on the TEPIC scores did not significantly impact the accuracy of the approach in most cases, especially for the scaled versions (Fig 3B). Hence, as for the ChIP-based model, the TEPIC score model seems to mainly capture the level of chromatin opening rather than the TF combinations responsible for gene expression. Conversely, our model solely built on DNA sequence features is not influenced by the chromatin accessibility and thus can yield relevant combinations of explanatory features (see the randomized control in Fig 3A and 3B). Note that the non-scaled version of TEPIC did show a loss of accuracy for cell-line H1-hESC (as well as a moderate loss for K562, but none for GM12878) when randomizing or maximizing the variables (Fig 3B). This result indicates that, although taking the abundance of open-chromatin peaks in the analyzed sequences does increase expression prediction accuracy, it might generate more irrelevant combinations of explanatory features than non-scaled versions.

### Contribution of additional genomic regions

Additional genomic regions were integrated into our model. We first thought to consider enhancer sequences implicated in transcriptional regulation. We used the enhancer mapping made by the FANTOM5 project, which identified 38,554 human enhancers across 808 samples [7]. This mapping uses the CAGE technology, which captures the level of activity for both promoters and enhancers in the same samples. It is then possible to predict the potential target genes of the enhancers by correlating the activity levels of these regulatory regions over hundreds of human samples [7]. However FANTOM5 enhancers are only assigned to 11,359 genes from the TCGA data, which correspond to the most expressed genes across different cancers (S5 Fig). Provided that the detection of enhancers relies on their activity, it is expected that enhancers are better characterized for the most frequently expressed genes. Because considering only the genes with annotated enhancers would considerably reduce the number of genes and including enhancers features only when available would introduce a strong bias in the performance of our model, we decided not to include these regulatory regions.

Second we analyzed the contribution of regions defined at the RNA level, namely 5’UTR, CDS, 3’UTR and introns, which can be responsible for post-transcriptional regulations [13, 17, 26, 42–50] (Fig 1). For all genes, we extracted all annotated 5’UTRs, 3’UTRs and CDSs, which were further merged and concatenated to a single 5’UTR, a single CDS, and a single 3’UTR per gene. Introns were defined as the remaining sequence (Fig 1). We also tested the potential contribution of the 1kb region located downstream the gene end, called thereafter Downstream Flanking Region (DFR, Fig 1). Our rationale was based on reports showing the presence of transient RNA downstream of polyadenylation sites [51], the potential presence of enhancers [7] and the existence of 5’ to 3’ gene looping [52].

**Fig 4:**
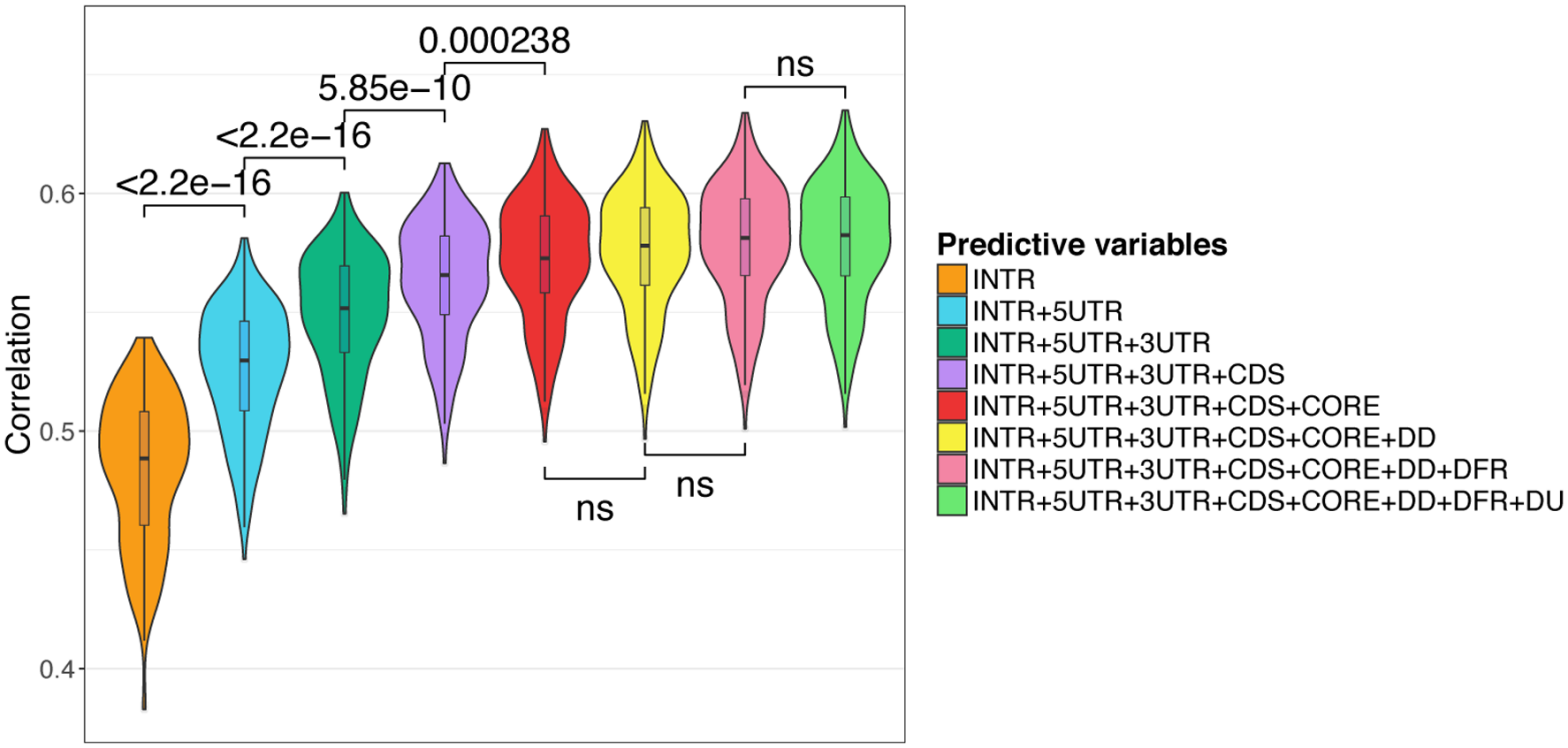
Contribution of additional genomic regions. Genomic regions were ranked according to their contribution in predicting gene expression. First, all regions were tested separately. Introns yielded the highest Spearman correlation between observed and predicted expressions (in a cross-validation procedure) and was selected as the ‘first’ seed region. Second, each region not already in the model was added separately. 5’UTR in association with introns yielded the best correlation and was therefore selected as the ‘second’ region. Third, the procedure was repeated till all regions were included in the model. The contribution of each region is then visualized starting from the most important (left) to the less important (right). Note that the distance between the second TSS and the first ATG is > 2000 bp for only 189 genes implying that 5’UTR and DD regions overlap. The correlations computed at each steps are indicated in (S2 Table). ns, non significant.

We used a forward selection procedure by adding one region at a time: (i) all regions were tested separately and the region leading to the highest Spearman correlation between observed and predicted expression was selected as the ‘first’ seed region, (ii) each region not already in the model was added separately and the region yielding the best correlation was selected (‘second region’), (iii) the procedure was repeated till all regions were included in the model. The correlations computed in a cross-validation procedure at each steps are indicated in S2 Table. As shown in Fig 4, the nucleotide composition of intronic sequences had the strongest contribution in the accuracy of our model, followed by UTRs (5’ then 3’) and CDS (Fig 4). The nucleotide composition of core promoter moderately increased the prediction accuracy. In contrast the composition of regions flanking core promoter (DU and DD, Fig 1) as well as regions located downstream the end of gene (DFR, Fig 1) did not significantly improve the predictions of our model. Note that combining all regions improved the performance of our model compared to promoter alone (compare Figs 2B and 4).

We compared models built on ssDNA and dsDNA, and ssDNA-based models yielded better accuracy S6 Fig. We also compared models built on percentages of nucleotides (n = 4), dinucleotides (n = 16) and nucleotides+dinucleotides (n = 20). As shown S7A Fig, dinucleotides provided stronger prediction accuracy than nucleotides and the best accuracy was obtained combining both nucleotides and dinucleotides. We also built a model on trinucleotide percentage (n = 64) (S7A Fig). This model did yield better results than model built on nucleotide+dinucleotide. However, the correlation increase was not as important as that observed when adding dinucleotides to nucleotides. Besides, the model built on trinucleotides involves more variables and is computationally demanding. We compared models built on nucleotides+dinucleotides adding individually trinucleotide percentages of each region (i.e. 8 models built on nucleotides+dinucleotides in all regions + trinucleotides in one specific region) (S7B Fig). This analysis revealed that the correlation increase observed when incorporating trinucleotides was mostly due to the contribution of trinucleotides computed in introns, reinforcing our conclusions regarding the importance of sequence-level instructions located in this region.

Because RNA-associated regions (introns, UTRs, CDSs) had greater contribution to the prediction accuracy compared to DNA regions (promoters, DFR), we compared the accuracy of our model in predicting gene vs. transcript expression. We retrieved the normalized results for gene expression (RNAseqV2 rsem.genes.normalized_results) and the matched normalized expression signal of individual isoforms (RNAseqV2 rsem.isoforms.normalized_results) for 225 TCGA samples. Accordingly, we generated a set a predictive variables specific to each isoform (see Material and methods). We found that models built on isoforms are less accurate than models built on genes (median r = 0.35, S8 Fig and (S3 Table)). Focusing on the broad nucleotide composition may not be optimal to model isoform expression and to differentiate expression of one isoform from another. Yet another simple explanation could be that reconstructing and quantifying full-length mRNA transcripts is a difficult task, and no satisfying solution exists for now [53]. Consequently isoform as opposed to gene expression is more difficult to measure and thus to predict.

### Additional validation of the model

In the above sections, our complete model, built on 160 variables corresponding to 4 nucleotide and 16 dinucleotide rates in 8 distinct regions (Fig 1), was trained with a data set containing 241 RNA-seq samples randomly chosen from 12 different cancers, and on 3 independent ENCODE RNA-seq datasets (see TEPIC comparison). We further evaluated our approach using two independent additional datasets: (a) a set of 1,270 RNA-seq samples collected from 14 cancer types and (b) a set of 582 microarray data. Overall, the RNA-seq and the microarray samples were collected from respectively 109 and 41 source sites and sequenced in 3 analysis centers. Similar accuracy was observed in all datasets (S9 and S10 Figs). Note that the correlations computed with microarray data were lower than that computed with RNA-seq data but involved lower number of genes (9,791 genes in microarrays vs. 16,294 in RNA-seq). For sake of comparison, we restricted RNA-seq data to the 9,791 microarray genes and we observed similar correlation (S10 Fig). Because our model was built on human reference genome, we also have computed the Spearman correlations between absolute values of CNV segment mean scores and model prediction errors calculated for each gene in 241 samples corresponding to 12 cancer types. The median correlation was -0.014, arguing against the model performance being related to CNV-density (S11 Fig).

### Selecting DNA features related to gene expression

**Fig 5:**
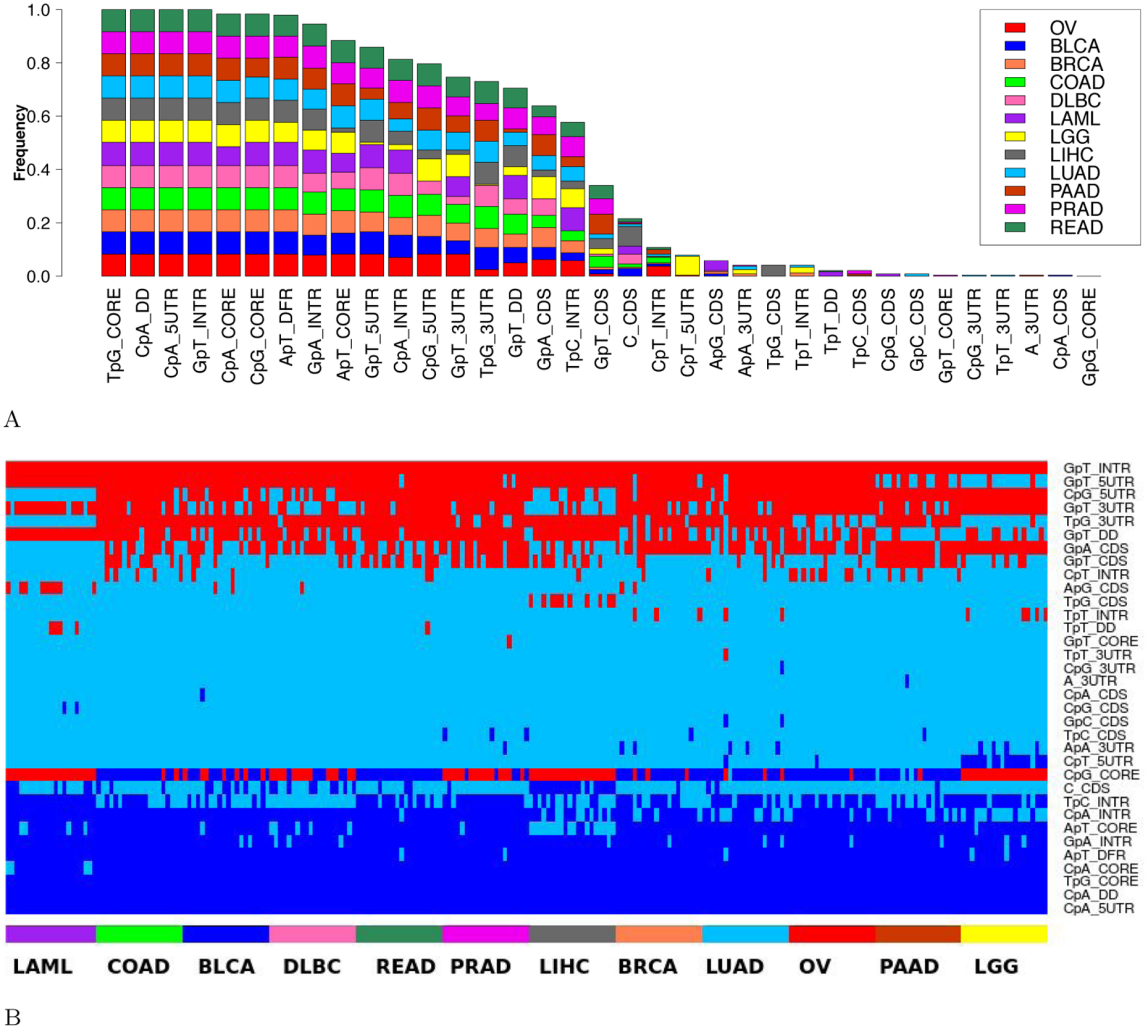
**A: Consistently selected variables among 12 types of cancer.** For each variable, the fraction of samples in which the variable is considered as stable (i. e. selected in more than 70% of the subsets after subsampling) is shown. Each color refers to a specific type of cancer. Only variables consistently selected in at least one sample are shown (out of the 160 variables). See Materials and methods for stable variable selection procedure and cancer acronyms. **B: Biological effect of the stable variables**. For each of the 241 samples (columns), a linear model was fitted using the variables (rows) stable for this sample only. The sign of the contribution of each variable in each sample is represented as follows: red for positive contribution, dark blue for negative contribution and sky blue refers to variables not selected (i.e. not stably selected for the considered sample). Only the variables stable in at least one sample are represented. Cancers and samples from the same cancer types are ranked by decreasing mean error of the linear model.

We sought the main DNA features related to gene expression. The complete model built on all 8 regions (160 variables) selected ∼ 129 predictive variables per sample. We used the stability selection algorithm developed by Meinshausen *et al.* [35] to identify the variables that are consistently selected after data subsampling (see Materials and methods for a complete description of the procedure). This procedure selected a median of ∼ 16 variables per sample. The barplot in Fig 5A shows, for each variable, the proportion of samples in which the variable is selected with high consistency (> 70% of the subsets).

We next determined whether stable variables exert a positive (activating) or a negative (inhibiting) effect on gene expression. For each sample, we fitted a linear regression model predicting gene expression using only the standardized variables that are stable for this sample. The activating/inhibiting effect of a variable is then indicated by the sign of its regression coefficient: < 0 for a negative effect and > 0 for a positive effect. The outcome of these analyses for all variables and all samples is shown Fig 5B. With the noticeable exception of CpG in the core promoter, all stable variables had an invariable positive (e.g. GpT in introns) or negative (e.g. CpA in DD and in 5UTR) contribution in gene expression prediction in all samples. In contrast, CpG in the core promoter had an alternating effect being positive in LAML and LGG for instance while negative in READ. It is also the only variable with a regression coefficient close to 0 (absolute value of median = 0.1, see S12 Fig), providing a partial explanation for the observed changes. As CpG methylation inhibits gene expression [38], we also investigated potential differences in core promoter methylation in LAML (positive contribution of CpG_CORE) and READ (negative contribution of CpG_CORE). We used the Illumina Infinium Human DNA Methylation 450 made available by TCGA and focused on the estimated methylation level (beta values) of the sites intersecting with the core promoter. We noticed that core promoters in LAML were overall more methylated (median = 0.85) than in READ (median = 0.69, wilcoxon test p-value < 2.2e-16), opposite to the sign of CpG coefficient in LAML (positive contribution of CpG_CORE) and READ (negative contribution of CpG_CORE). This argued against a contribution of methylation in the alternating effect of CpG_CORE.

**Fig 6:**
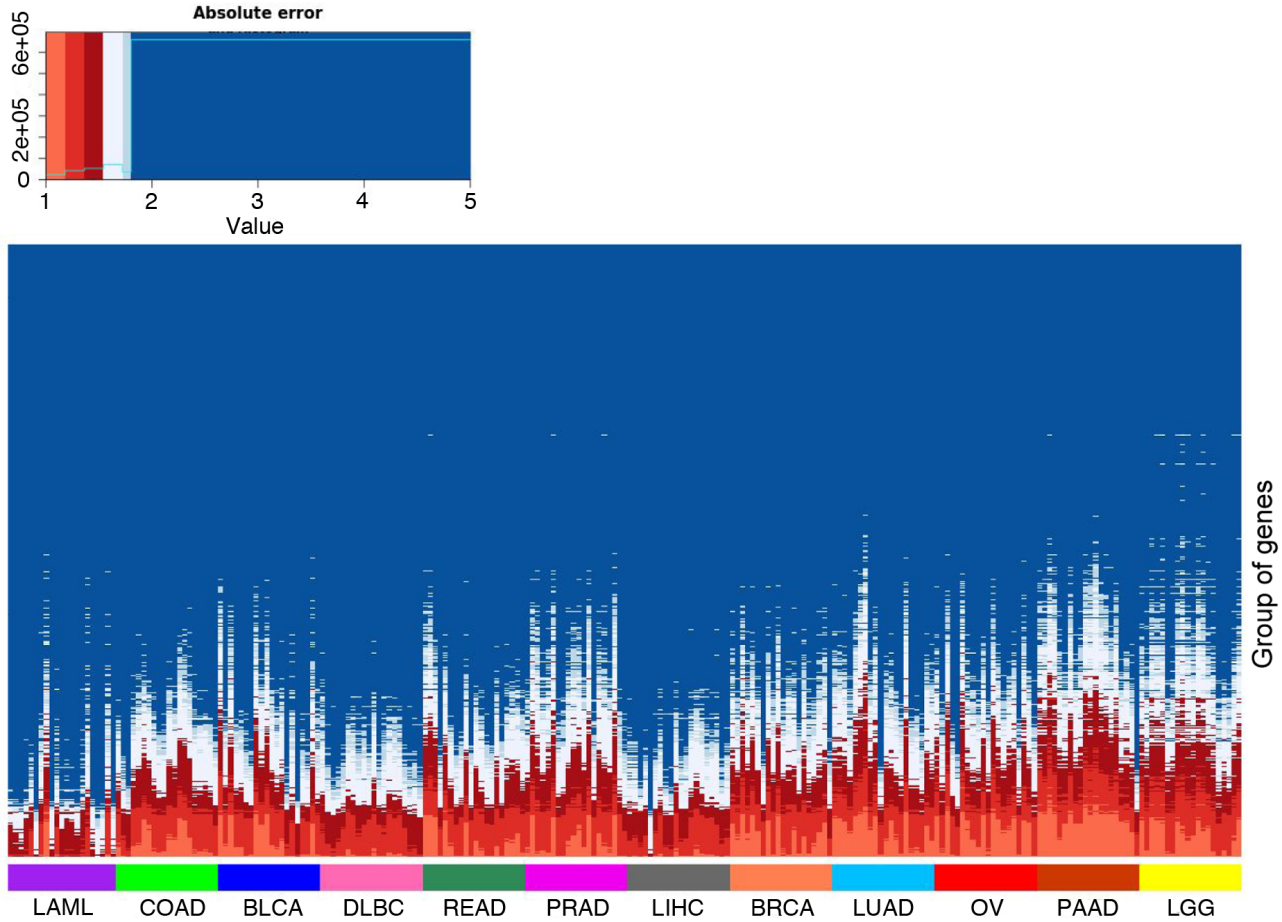
Gene classification according to prediction accuracy. Columns represent the various samples gathered by cancer type. Samples from the same cancer type are ranked by decreasing mean squared prediction error. Lines represent the 3,680 groups of gene obtained with the regression trees (one tree for each of the 241 samples) ranked by decreasing mean squared prediction error. Groups gathering the top 25% well predicted genes (error <∼ 1.77) are indicated in red and light blue.

We observed that the accuracy of our model varied between cancer types (S9 Fig). In order to characterize well predicted genes in each sample, we used a regression tree [54] to classify genes according to the prediction accuracy of our model (i.e. absolute error). The nucleotide and dinucleotide compositions of the various considered regions were used as classifiers. This approach identified groups of genes with similar (di)nucleotide composition in the regulatory regions considered and for which our model showed similar accuracy (S13 Fig). Implicitly, it identified the variables associated with a better or a poorer prediction. We applied this approach to the 241 linear models. The number of groups built by a regression tree differs from one sample to another (average number = 14). The resulting 3,680 groups can be visualized in the heatmap depicted in Fig 6, wherein each column represents a sample and each line corresponds to a group of genes identified by a regression tree. This analysis showed that our model is not equally accurate in predicting the expression of all genes but mainly fits certain classes of genes (bottom rows of the heatmap, Fig 6) with specific genomic features (S13 Fig). Note that the groups well predicted in all cancers presumably correspond to highly and ubiquitously expressed housekeeping genes: groups with low prediction error in all samples and cancer types (see S13 Fig for an example group of 996 genes identified by a regression tree learned in one PRAD sample) are functionally enriched for general and widespread biological processes (S4 Table). In contrast, groups well predicted in only certain cancers were associated to specific biological function. For instance, a regression tree learned on one PAAD sample identified a group of 1,531 genes, which has low prediction error in LGG and PAAD samples but high error in LAML, LIHC and DLBC samples (Fig 6 and S13 Fig). Functional annotation of this group showed that, in contrast to the group described above (S13 Fig and S4 Table), this group is also linked to specific biological processes (S5 Table).

We further computed Gini coefficient for 16,134 genes using 8,556 GTEx libraries [55]. Gini coefficient measures statistical dispersion which can be used to measure gene expression ubiquity: value 0 represents genes expressed in all samples, while value 1 represents genes expressed in only one sample. We observed that the correlations obtained between Gini coefficient and model errors in each TCGA sample ranged from 0.22 to 0.36. We also compared model errors associated to first and last quartiles of the Gini coefficient distribution using a Wilcoxon test for each of the 241 samples. The test was invariably significant with maximum p-value = 2.881*e*^−7^. Likewise analyses were performed with 1,897 FANTOM CAGE libraries [56] considering 15,904 genes. In that case, correlation between models errors and Gini coefficients ranged from 0.25 to 0.4. Overall these analyses suggested that our model better predicts expression of highly and ubiquitously expressed genes. We do not exclude that, when predicting tissue-specific genes, ChIP-seq data collected from the same tissue may add explanatory power to the sequence model. Note, however, that the model performances vary between cancer and cell types implying that part of cell-specific genes are also well predicted by the model (S9 Fig).

### Relationships between selected nucleotide composition and genome architecture

**Fig 7:**
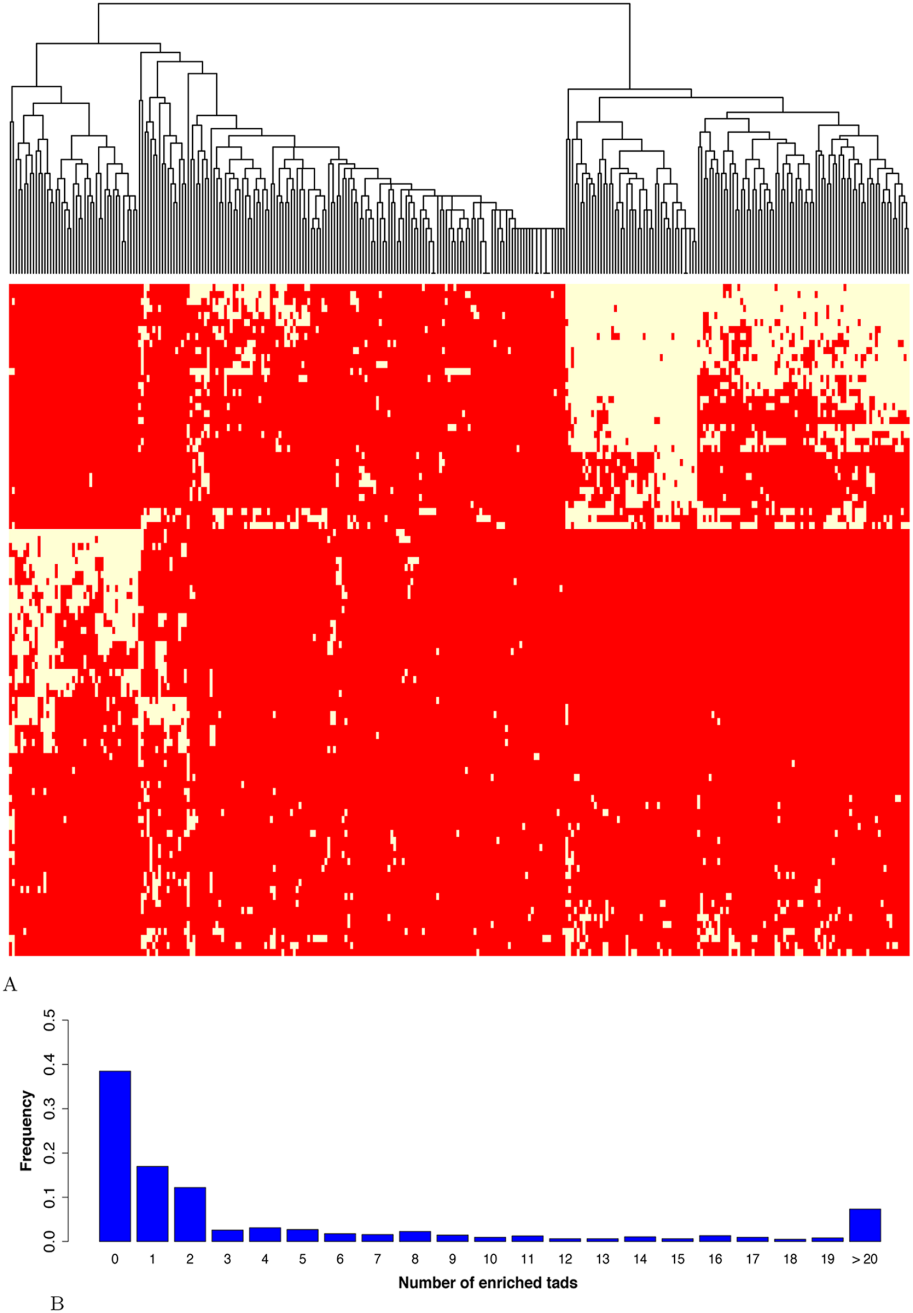
**A: Nucleotide compositions of resident genes distinguish TADs.** For each TAD and for each region considered, the percentage of each nucleotide and dinucleotide associated to the embedded genes were compared to that of all other genes using a Kolmogorov-Smirnov test. Red indicates FDR-corrected p-value ≥ 0.05 and yellow FDR-corrected p-value < 0.05. TAD clustering was made using this binary information. Only TADs with at least one p-value < 0.05 are shown (i.e. 87% of the TADs containing at least 10 genes). y-axis from top to bottom: G_INTR, GpC_INTR, CpC_INTR, CpC_3UTR, GpC_3UTR, G_3UTR, GpC_CDS, CpC_CDS, G_CDS, G_DFR, CpC_DFR, GpC_DFR, CpG_INTR, CpG_3UTR, CpG_CDS, CpG_DFR, G_DU, GpC_DD, CpG, DU, CpG_DD, GpC_DU, CpC_DU, CpC_DD, G_DD, GpC_5UTR, CpG_5UTR, G_5UTR, GpC_CORE, CpG_CORE, CpC_CORE, G_CORE, CpC_5UTR, CpT_3UTR, CpT_CDS, CpT_INTR, ApT_INTR, TpA_INTR, A_INTR, ApA_INTR, TpA_3UTR, ApT_3UTR, A_3UTR, ApA_3UTR, ApA_CDS, A_CDS, ApT_CDS, TpA_CDS, A_DD, ApA_DD, ApT_DD, TpA_DD, TpA_DU, ApT_DU, ApA_DU, A_DU, TpA_DFR, ApT_DFR, A_DFR, ApA_DFR, ApA_CORE, A_CORE, ApT_CORE, TpA_CORE, ApA_5UTR, ApT_5UTR, A_5UTR, TpA_5UTR, ApC_DFR, ApC_DD, ApC_DU, TpC_DU, TpC_DFR, ApC_CORE, CpA_DU, CpA_DFR, CpA_CDS, ApC_CDS, ApC_3UTR, TpC_CDS, TpC_CORE, CpT_5UTR, TpC_5UTR, CpT_CORE, TpC_DD, CpA_CORE, ApC_5UTR, CpA_5UTR, ApC_INTR, CpA_DD, CpT_DFR, CpT_DD, CpT_DU, TpC_3UTR, TpC_INTR, CpA_INTR, CpA_3UTR. **B: TAD enrichment within groups of genes whose expression is accurately predicted by our model.** The enrichment for each TAD (containing more than 10 genes) in each gene group accurately predicted by our model (i.e. groups with mean error < mean errors of the 1st quartile) was evaluated using an hypergeometric test. The fraction of groups with enriched TADs (p-value < 0.05) is represented.

We probed the regulatory activities of the selected regions. We first determined whether introns contained specific regulatory sequence code by assessing the presence of *cis* expression quantitative trait loci (*cis*-eQTLs). Zhou *et al.* indeed showed that the effect of eQTL SNPs can be predicted from a regulatory sequence code learned from genomic sequences [25]. These findings also implied that *cis*-eQTLs preferentially affect DNA sequences at precise locations (e.g. TF binding sites) rather than global nucleotide composition (i.e. nucleotide/dinucleotide percentages used as variables in our model). We used the v6p GTEx release to compute the average frequencies of *cis*-eQTLs present in the considered genomic regions and directly linked to their host genes (S6 Table). We noticed that introns contained the smallest density of *cis*-eQTLs (10 times less than any other regions), while containing comparable amount of SNPs (S7 Table). This result argued against the presence of a regulatory sequence code similar to that observed in promoters for instance [25], despite the presence of enhancers (S8 Table. These results rather unveiled the existence of another layer of intron-mediated regulation, which involves global nucleotide compositions of larger DNA regions. We then asked whether the groups of genes identified by the regression trees (Fig 6) correspond to specific TADs. Genes within the same TAD tend to be coordinately expressed [57, 58]. TADs with similar chromatin states tend to associate to form two genomic compartments called A and B: A contains transcriptionally active regions while B corresponds to transcriptionally inactive regions [59]. The driving forces behind this compartmentalization and the transitions between compartments observed in different cell types are not fully understood, but chromatin composition and transcription are supposed to play key roles [5]. Jabbari and Bernardi showed that nucleotide composition along the genome (notably isochores) can help define TADs [60]. As intronic sequences represent ∼ 50% of the human genome (1,512,685,844 bp out of 3,137,161,264 according to ENSEMBL merged intron coordinates), the nucleotide composition of introns likely resemble that of neighbor genes and more globally that of the corresponding TAD. We used the 373 TADs containing more than 10 genes mapped in IMR90 cells [6]. For each TAD and each (di)nucleotide, we used a Kolmogorov-Smirnov test to compare the (di)nucleotide distribution of the embedded genes with that of all other genes. We used a Benjamini-Hochberg multiple testing correction to control the False Discovery Rate (FDR), which was fixed at 0.05 (see Materials and methods section). We found that 324 TADs out of 373 (∼87%) are characterized by at least one specific nucleotide signature (Fig 7A). In addition, our results clearly showed the existence of distinct classes of TADs related to GC content (GC-rich, GC-poor and intermediate GC content) (Fig 7A), in agreement with [60]. We next considered the 967 groups of genes defined in Fig 6 whose expression is accurately predicted by our model (i.e. groups with mean error < mean error of the 1st quartile). We thus focused our analyses on genes for which we did learn some regulatory features. We evaluated the enrichment for specific TADs in each group (considering only TADs containing more than 10 genes) using an hypergeometric test (Fig 7B). We found that 60% of these groups were enriched for at least one TAD (p-value < 0.05). Hence, several groups of genes identified by the regression trees (Fig 6) do correspond to specific TADs (Fig 7B). We concluded that our model, primarily based on intronic sequences, select gene nucleotide compositions that better distinguish active TADs.

## Discussion

In this study, we corroborate the hypothesis that DNA sequence contains information able to explain gene expression [20–25]. We built a global regression model to predict, in any given sample, the expression of the different genes using only nucleotide compositions as predictive variables. Overall our model provided a framework to study gene regulation, in particular the influence of regulatory regions and their associated nucleotide composition.

A surprising result of our study is that sequence-level information is highly predictive of gene expression and in some occasions comparable to reference ChIP-seq data alone [17, 19]. The similar accuracy of models built on real and randomly permuted experimental data indicated that, though the experimental data are biologically relevant, their interpretation through a linear model, in particular inference of TF combinations, is not straightforward as randomization of experimental data did not show the expected loss of accuracy (Fig 3). An interesting perspective would be to devise a strategy to infer TF combinations from experimental data without being influenced by the opening of the chromatin.

The accuracy of our model confirmed that DNA sequence *per se* and basic information like dinucleotide frequencies have very high predictive power. It remains to determine the exact nature of these sequence-level instructions. Interestingly, nucleotide environment contributes to prediction of TF binding sites and motifs bound by a TF have a unique sequence environment that resembles the motif itself [40]. Hence, the potential of the nucleotide content to predict gene expression may be related to the presence of regulatory motifs and TFBSs. However, we showed that the gene body (introns, CDS and UTRs), as opposed to sequences located upstream (promoter) or downstream (DFR), had the most significant contribution in our model. Moreover, *cis*-eQTL frequencies argue against the presence of a regulatory sequence code in introns similar to that observed in promoters, suggesting the existence of another layer of regulation implicating the nucleotide composition of large DNA regions.

Gene nucleotide compositions vary across the genome and can even help define TAD boundaries [60]. In line with [60], we showed that genes located within the same TAD share similar nucleotide compositions, which provides a nucleotide signature for their TADs (Fig 7A). Our model aimed at predicting gene expression, and therefore intimately linked to TAD compartmentalization, appeared to capture these signatures. Several studies have already demonstrated the existence of sequence-level instructions able to determine genomic interactions. Using an SVM-based approach, Nikumbh *et al* demonstrated that sequence features can determine long-range chromosomal interactions [61]. Similar results were obtained by Singh *et al.* using deep learning-based models [62]. Using biophysical approaches, Kornyshev *et al.* showed that sequence homology influences physical attractive forces between DNA fragments [63]. It would be interesting to determine whether the nucleotide signatures identified by our model are directly implicated in DNA folding and 3D genome architecture.

Finally, although sequence-level instructions are—almost—identical in all cells of an individual, their usage must be cell-type specific to allow proper A/B compartimentalization of TADs, gene expression and ultimately diversity of cell functions. At this stage, the mechanisms driving this cell-type specific selection of nucleotide compositions remain to be characterized.

## Supporting information

S1 Fig Comparison of models built on maximum or sum PWM motif scores. The model was built (i) using 60 nucleotide/dinucleotide percentages computed in the 3 promoter segments (CORE+DU+DD) and 471 JASPAR2016 PWM maximum scores computed in the CORE segment (pink) or (ii) using 60 nucleotide/dinucleotide percentages computed in the 3 promoter segments (CORE+DU+DD) and 471 JASPAR2016 PWM sum scores computed in the CORE segment (green). All sequences were centered around the 2nd TSS and the 2 models were fitted on 16,294 genes for each of the 241 samples.
(PDF)

S2 Fig Dinucleotide local distribution around GENCODEv24 TSSs. Dinucleotide percentages (y-axis) along 140,604 DNA regions centered around GENCODE v24 TSSs ±2000 bp (the distance to TSS is shown in the x-axis). Dinucleotide combinations are represented as first nucleotide on left and second nucleotide on top. The promoter segmentation used in this study (Fig 1) is indicated with vertical dashed lines at -500 bp and 500 bp from the TSS.
(PDF)

S3 Fig Number of TSSs by gene. We considered 19,393 TCGA genes listed in TCGA and the TSSs annotated by GENCODE v24.
(PDF)

S4 Fig Contribution in the model of the TSS number. The model is built using 20 variables corresponding to the nucleotide (4) and dinucleotide (16) percentages computed in the CORE promoter (red), DU (green) or DD (yellow) centered around the second TSS as predictive variables (green). Linear models are also built on the number of isoforms (dark pink) and the number of TSSs (dark blue). Finally models are built using the combinations of variables indicated. All different models were fitted on 19,393 genes for each of the 241 samples considered. The prediction accuracy was evaluated in each sample by evaluating the Spearman correlation coefficients between observed and predicted gene expressions. The correlations obtained in all samples are shown as violin plots. These two last plots underscored the importance of these two variables in predicting gene expression.
(PDF)

S5 Fig Gene expression distribution and FANTOM5 enhancer association. The 19,393 genes listed in one LAML sample (TCGA.AB.2939.03A.01T.0740.13_LAML) (pink) and a subset of 11,359 genes with assigned FANTOM enhancers (green) were considered. The median expression of genes with assigned enhancers is greater than that of all genes (wilcoxon test p-value < 2.2e-16)
(PDF)

S6 Fig Accuracies of models built on dsDNA or ssDNA. **A:** Models were built using nucleotide and dinucleotide percentages computed on dsDNA (2 nucleotides + 8 dinucleotides; green violin) or on ssDNA (4 nucleotides + 16 dinucleotides; purple violin) in all the regulatory regions (CORE, DU, DD, 5UTR, CDS, 3UTR, INTR, DFR). The 2 models were fitted on 16,294 genes for each of the 241 samples. The prediction accuracy was evaluated in each sample by evaluating the Spearman correlation coefficients. **B:** Same analyses focusing on each of the indicated regions.
(PDF)

S7 Fig Model accuracy with different set of nucleotide predictive variables. **A:** Models were built using different set of variables including nucleotide (4 x 8 regions), dinucleotide (16 x 8 regions) and/or trinucleotide (64 x 8 regions) percentages computed in all the regulatory regions (CORE, DU, DD, 5UTR, CDS, 3UTR, INTR, DFR). All different models were fitted on 16,280 genes for each of the 241 samples considered. The prediction accuracy was evaluated in each sample by evaluating the Spearman correlation coefficients. **B:** Models were built using nucleotide (4 x 8 regions) and dinucleotide (16 x 8 regions) percentages computed in all the regulatory regions and trinucleotide (64) percentages computed in each of the indicated region separately.
(PDF)

S8 Fig Forward selection procedure with models built on isoform expressions. The procedure is identical to that described in Fig 4 but models were built on isoform-specific variables and correlations were computed between observed and predicted isoform expression, not gene expression.
(PDF)

S9 Fig Model accuracy in different cancer types. The model with 160 variables (20 (di)nucleotide rates in 8 regions) was built on 16,294 genes in 241 samples corresponding to the initial training set corresponding to 12 cancer types (**A**) and in an additional set of 1,270 samples corresponding to 14 different cancer types (**B**). The prediction accuracy was evaluated in each sample by evaluating the Spearman correlation coefficients between observed and predicted gene expressions. The correlations obtained in all samples of each data sets are shown as violin plots in **A** (training set) and **B** (additional set). The color code indicates the cancer types. The horizontal dashed lines indicates the median correlation (**A**, 0.582; **B**, 0.577).
(PDF)

S10 Fig Comparison on models built on RNA-seq or microarray data. The model with 160 variables (20 (di)nucleotide rates in 8 regions) was built on 9,791 genes in 582 samples with matched RNA-seq and microarray data. The prediction accuracy was evaluated in each sample by evaluating the Spearman correlation coefficients between observed and predicted gene expressions. The correlations obtained in all samples with RNA-seq- or microarray-built models are shown as violin plots.
(PDF)

S11 Fig Spearman correlations between CNV segment mean score and model prediction error. CNV absolute segment mean scores were computed for each as explained in Materials and Methods section. Model prediction absolute error for each gene are given by our predictive model using nucleotide and dinuclotide percentages computed in all the regulatory regions. Models were fitted on 16,294 genes for each of the 234 on 241 samples having CNV TCGA data available. The median correlation for the 234 samples is -0.014.
(PDF)

S12 Fig Absolute values of the regression coefficients. A linear regression model was built, for each sample, on standardized stable variables only. The boxplots show absolute values of the corresponding coefficients in all samples for each variable considered. Color code as in Fig 5. CpG in the core promoter is highlighted in white. Purple line represents the median of CpG_CORE coefficients.
(PDF)

S13 Fig Example of regression trees learned on two linear models. **A: Regression tree leading to a group of genes well predicted in all samples.** This tree has been learned on the sample TCGA.FC.A5OB.01A.11R.A29R.07_PRAD using all nucleotide composition in all regions. The red path defines a group of 996 genes which has low Lasso error in all samples and cancer types. This group was used for functional annotation (S4 Table). **B: Regression tree leading to a group of genes well predicted in LGG and PPAD samples.** This tree has been learned on the sample TCGA.IB.7646.01A.11R.2156.07_PAAD using all nucleotide composition in all regions. The red path defines a group of 1,531 genes which has low Lasso error in LGG and PAAD samples but high error in LAML, LIHC and DLBC samples. This group was used for functional annotation (S5 Table).
(PDF)

S1 Table Model comparison. Each model is fitted for each tumor, using all the variables over all regions (160 variables among 8 regulatory regions). First and second columns are median correlation and mean square error over all the tumors. The third column represents mean computing time per tumor (in minutes) on a standard laptop.
(PDF)

S2 Table Contributions of additional genomic regions. Genomic regions were ranked according to their contribution in predicting gene expression. First, all regions were tested separately. Introns yielded the highest Spearman correlation between observed and predicted expressions and was selected as the ‘first’ seed region. Second, each region not already in the model was added separately. 5UTR in association with introns yielded the best correlation and was therefore selected as the ‘second’ region. Third, the procedure was repeated till all regions were included in the model. The contribution of each region is then visualized starting from the most important (left) to the less important (right). The correlations computed at each steps are indicated.
(PDF)

S3 Table Correlations between observed and predicted isoform expression. The procedure is identical to that described in S2 Table but models were built on isoform-specific variables and correlations were computed between observed and predicted isoform expression, not gene expression.
(PDF)

S4 Table Functional enrichment of a group of genes well predicted in all samples. The group of 996 genes is obtained by fitting a regression tree on the sample TCGA.FC.A5OB.01A.11R.A29R.07_PRAD using all the nucleotide composition in all regions. These genes are well predicted (mean error < 1st quartile) for all samples of different type cancers. This group of genes was further annotated using the DAVID functional annotation tool. Only the top 5 biological processes indicated by DAVID is shown. The GO term yielded by this analysis corresponded to general and widespread biological processes indicating that these genes likely corresponded to housekeeping genes.
(PDF)

S5 Table Functional enrichment of a group of genes well predicted in LGG and PAAD. The group of 1,531 genes is obtained by fitting a regression tree on the sample TCGA.IB.7646.01A.11R.2156.07_PAAD using all the nucleotide composition in all regions. These genes are well predicted (mean error < 1st quartile) for all LGG and PAAD samples but not that of LAML, DBLC and LIHC. This group of genes was further annotated using the DAVID functional annotation tool. Only the top 5 biological processes indicated by DAVID is shown. The GO term “Nervous system development” indicates that these genes can be involved in specific biological processes.
(PDF)

S6 Table Frequencies of *cis*-eQTLs in the genomic regions considered. We computed the density of *cis*-eQTL per regulatory region by dividing the sum of *cis*-eQTLs intersecting with the region considered for all genes by the sum of the lengths of the same regulatory region of all genes. see Material and methods for details.
(PDF)

S7 Table Frequencies of SNPs in CORE and INTRON regions. We computed the density of SNPs per regulatory region by dividing the sum of SNPs intersecting with the region considered for all genes by the sum of the lengths of the same regulatory region of all genes. We only considered SNPs detected on chromosomes 1, 2 and 19. see Material and methods for details.
(PDF)

S8 Table Intersection between enhancers and the genomic regions considered. We computed the density of enhancers per regulatory region by dividing the total length of the intersection between the enhancers and the region considered for all genes by the sum of the lengths of the same regulatory region of all genes. see Material and methods for details.
(PDF)
