# Supplementary figures and images for: Probing instructions for expression regulation in gene nucleotide compositions
Source: PLoS Comput Biol. 2018 Jan 2;14(1):e1005921. doi: 10.1371/journal.pcbi.1005921 (PMC5766238; doi:10.1371/journal.pcbi.1005921)

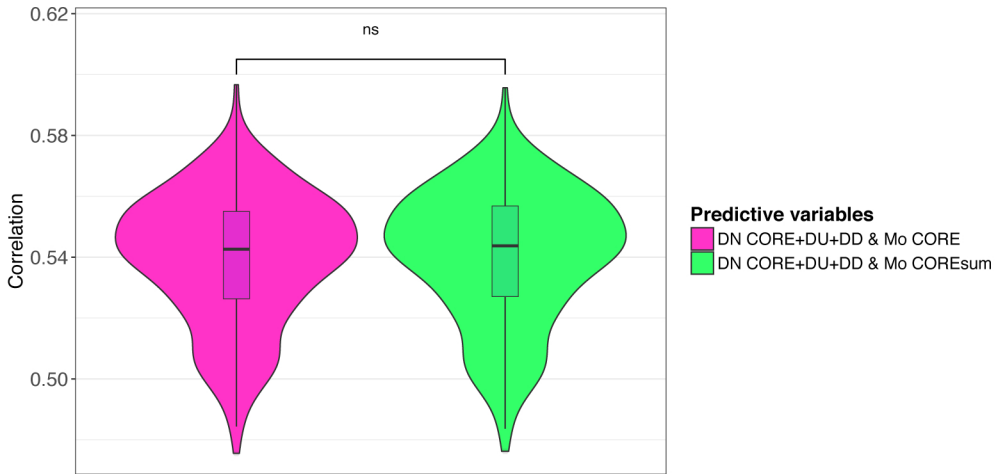

Supplement: S1 Fig — The model was built (i) using 60 nucleotide/dinucleotide percentages computed in the 3 promoter segments (CORE+DU+DD) and 471 JASPAR2016 PWM maximum scores computed in the CORE segment (pink) or (ii) using 60 nucleotide/dinucleotide percentages computed in the 3 promoter segments (CORE+DU+DD) and 471 JASPAR2016 PWM sum scores computed in the CORE segment (green). All sequences were centered around the 2nd TSS and the 2 models were fitted on 16,294 genes for each of the 241 samples. (PDF) [file pcbi.1005921.s001.pdf]

$-A$  $-C$  $-G$  $-T$ 

A-

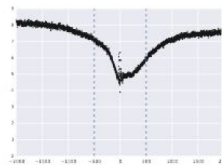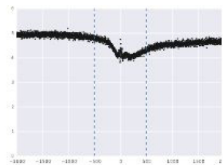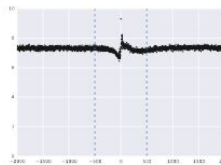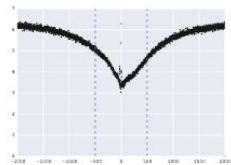

C-

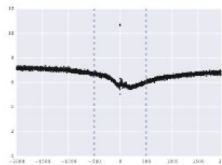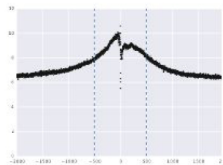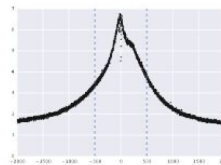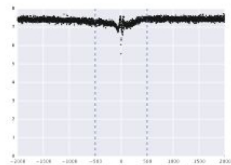

G-

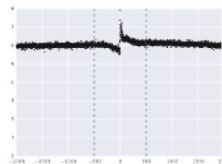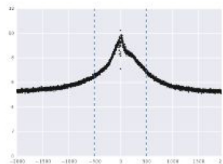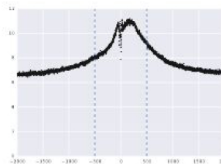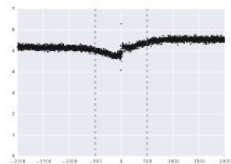

T-

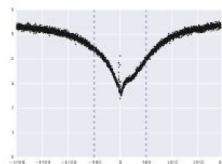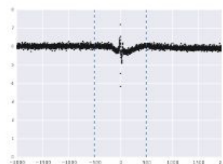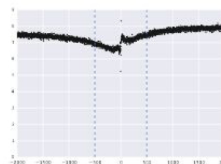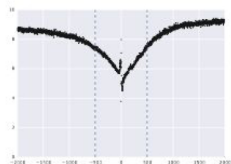

Supplement: S2 Fig — Dinucleotide percentages (y-axis) along 140,604 DNA regions centered around GENCODE v24 TSSs ±2000 bp (the distance to TSS is shown in the x-axis). Dinucleotide combinations are represented as first nucleotide on left and second nucleotide on top. The promoter segmentation used in this study (Fig 1) is indicated with vertical dashed lines at -500 bp and 500 bp from the TSS. (PDF) [file pcbi.1005921.s002.pdf]

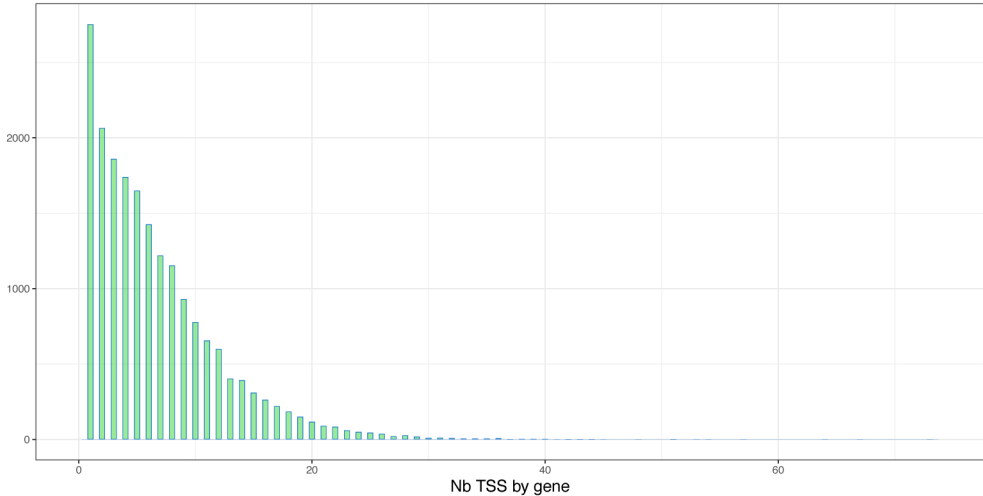

Supplement: S3 Fig — We considered 19,393 TCGA genes listed in TCGA and the TSSs annotated by GENCODE v24. (PDF) [file pcbi.1005921.s003.pdf]

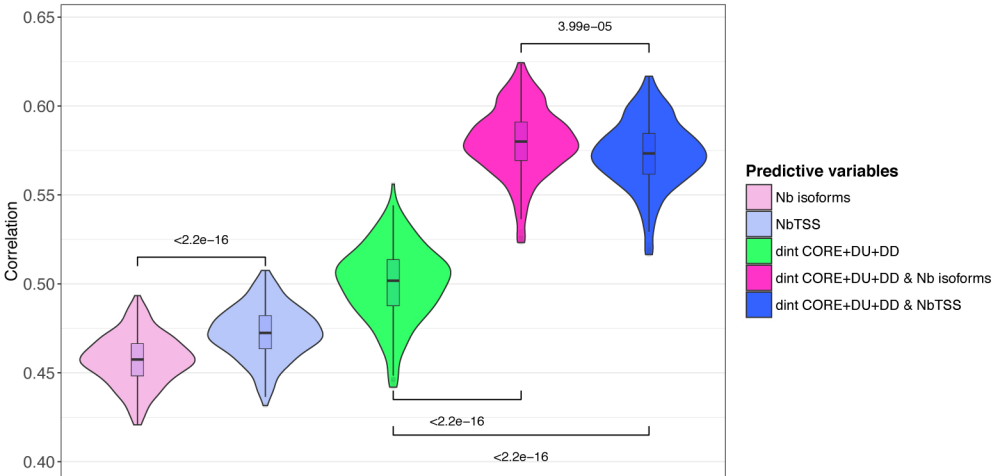

Supplement: S4 Fig — The model is built using 20 variables corresponding to the nucleotide (4) and dinucleotide (16) percentages computed in the CORE promoter (red), DU (green) or DD (yellow) centered around the second TSS as predictive variables (green). Linear models are also built on the number of isoforms (dark pink) and the number of TSSs (dark blue). Finally models are built using the combinations of variables indicated. All different models were fitted on 19,393 genes for each of the 241 samples considered. The prediction accuracy was evaluated in each sample by evaluating the Spearman correlation coefficients between observed and predicted gene expressions. The correlations obtained in all samples are shown as violin plots. These two last plots underscored the importance of these two variables in predicting gene expression. (PDF) [file pcbi.1005921.s004.pdf]

AML – wilcoxon test : p-value < 2.2e-16

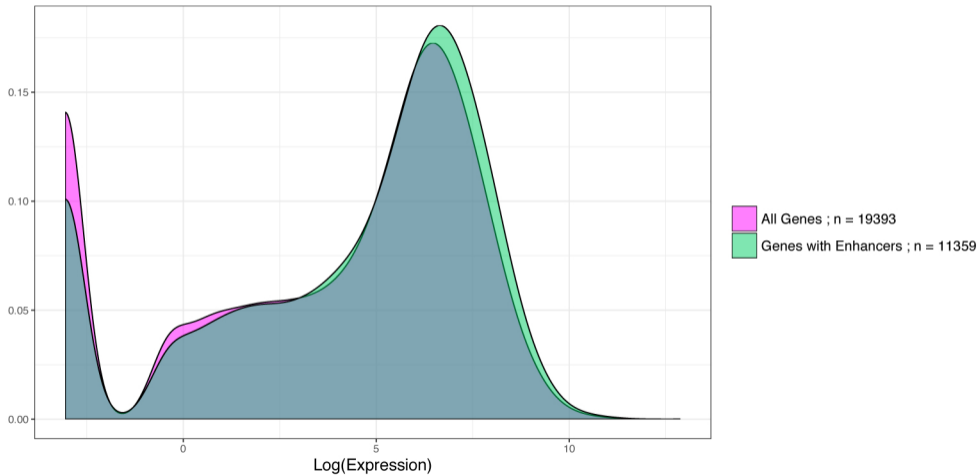

Supplement: S5 Fig — The 19,393 genes listed in one LAML sample (TCGA.AB.2939.03A.01T.0740.13_LAML) (pink) and a subset of 11,359 genes with assigned FANTOM enhancers (green) were considered. The median expression of genes with assigned enhancers is greater than that of all genes (wilcoxon test p-value < 2.2e-16) (PDF) [file pcbi.1005921.s005.pdf]

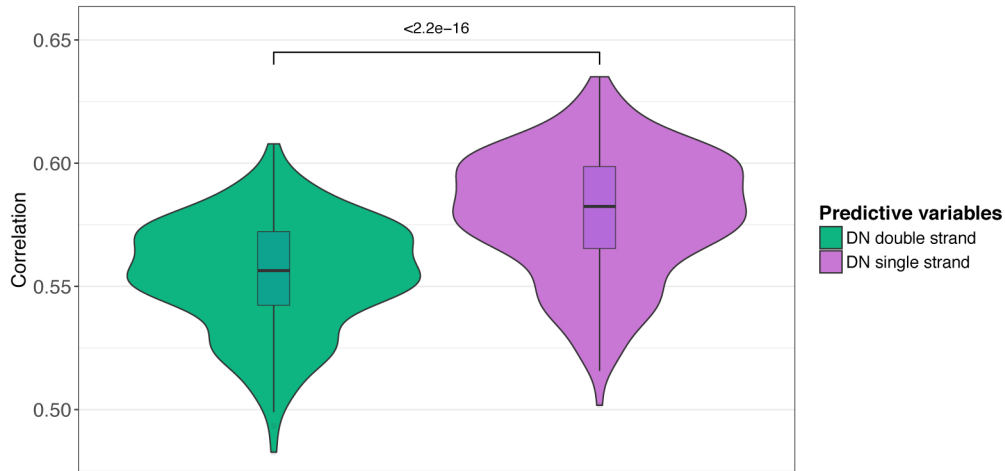

A

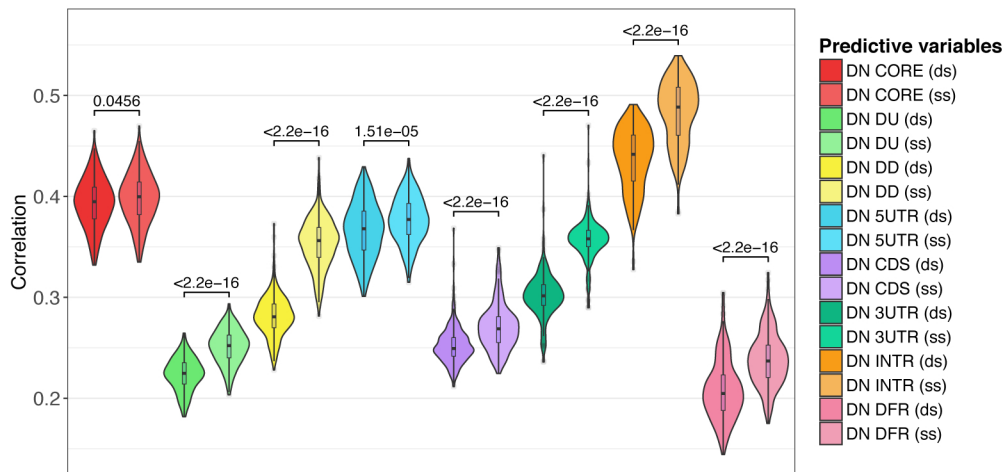

B

Supplement: S6 Fig — A: Models were built using nucleotide and dinucleotide percentages computed on dsDNA (2 nucleotides + 8 dinucleotides; green violin) or on ssDNA (4 nucleotides + 16 dinucleotides; purple violin) in all the regulatory regions (CORE, DU, DD, 5UTR, CDS, 3UTR, INTR, DFR). The 2 models were fitted on 16,294 genes for each of the 241 samples. The prediction accuracy was evaluated in each sample by evaluating the Spearman correlation coefficients. B: Same analyses focusing on each of the indicated regions. (PDF) [file pcbi.1005921.s006.pdf]

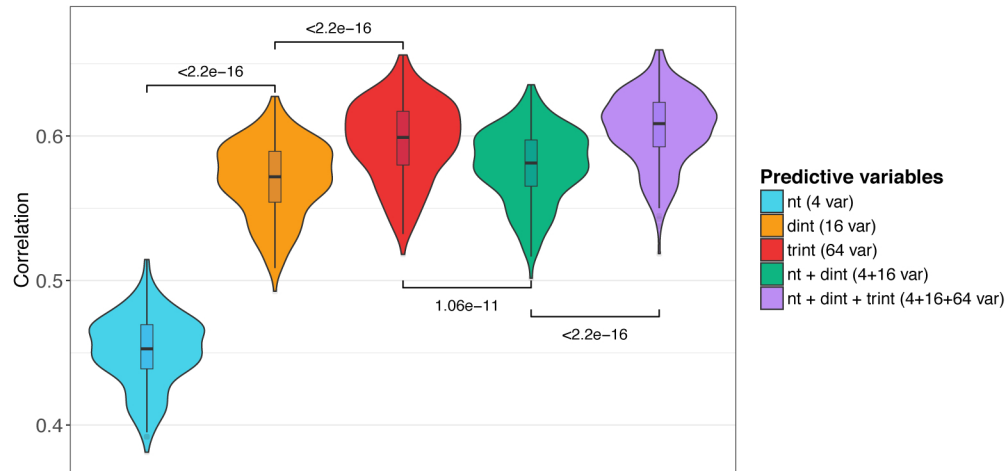

A

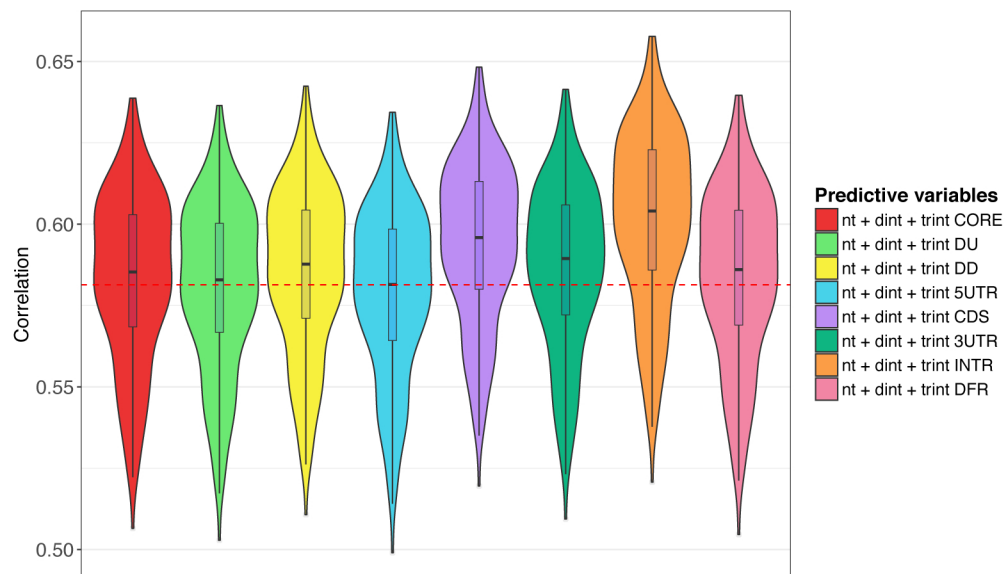

B

Supplement: S7 Fig — A: Models were built using different set of variables including nucleotide (4 x 8 regions), dinucleotide (16 x 8 regions) and/or trinucleotide (64 x 8 regions) percentages computed in all the regulatory regions (CORE, DU, DD, 5UTR, CDS, 3UTR, INTR, DFR). All different models were fitted on 16,280 genes for each of the 241 samples considered. The prediction accuracy was evaluated in each sample by evaluating the Spearman correlation coefficients. B: Models were built using nucleotide (4 x 8 regions) and dinucleotide (16 x 8 regions) percentages computed in all the regulatory regions and trinucleotide (64) percentages computed in each of the indicated region separately. (PDF) [file pcbi.1005921.s007.pdf]

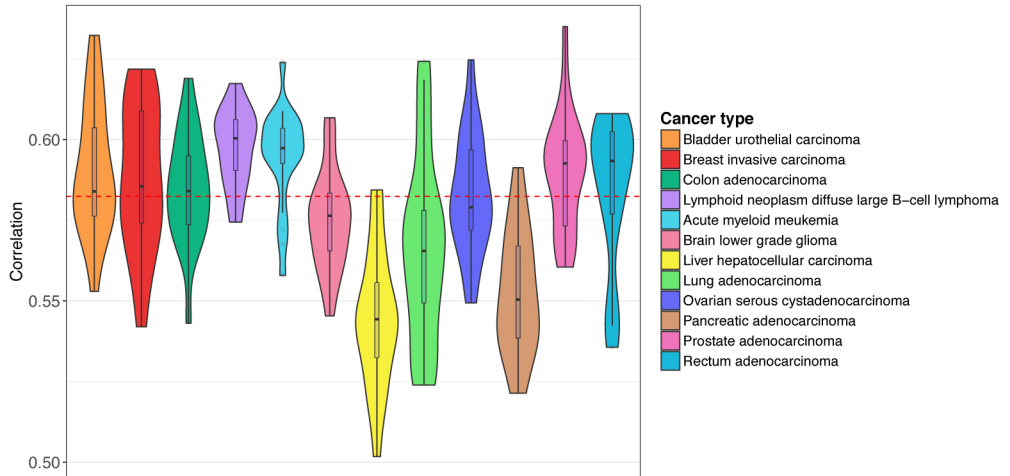

A

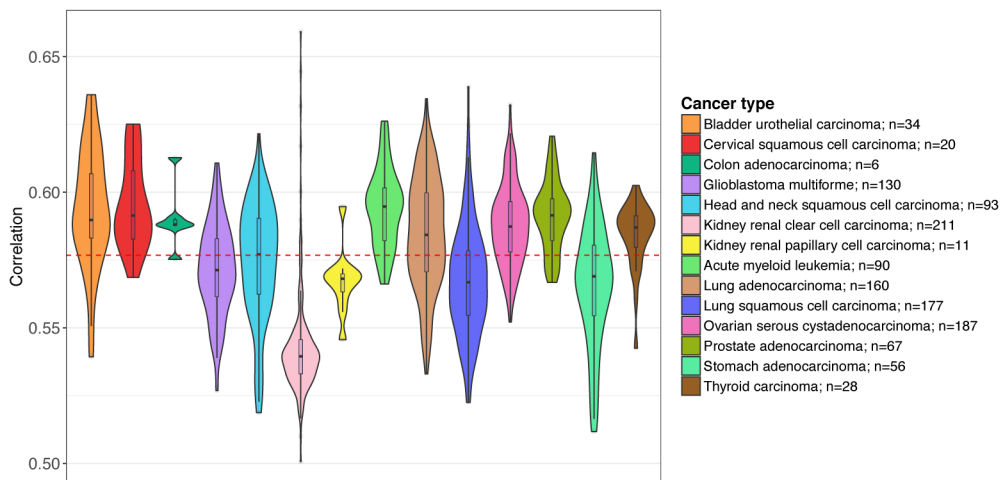

B

Supplement: S9 Fig — The model with 160 variables (20 (di)nucleotide rates in 8 regions) was built on 16,294 genes in 241 samples corresponding to the initial training set corresponding to 12 cancer types (A) and in an additional set of 1,270 samples corresponding to 14 different cancer types (B). The prediction accuracy was evaluated in each sample by evaluating the Spearman correlation coefficients between observed and predicted gene expressions. The correlations obtained in all samples of each data sets are shown as violin plots in A (training set) and B (additional set). The color code indicates the cancer types. The horizontal dashed lines indicates the median correlation (A, 0.582; B, 0.577). (PDF) [file pcbi.1005921.s009.pdf]

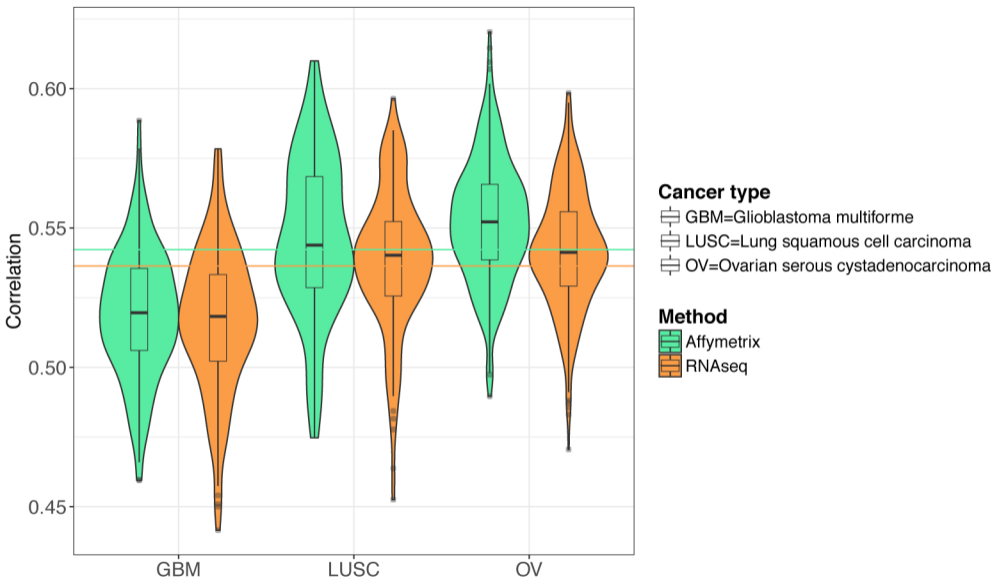

Supplement: S10 Fig — The model with 160 variables (20 (di)nucleotide rates in 8 regions) was built on 9,791 genes in 582 samples with matched RNA-seq and microarray data. The prediction accuracy was evaluated in each sample by evaluating the Spearman correlation coefficients between observed and predicted gene expressions. The correlations obtained in all samples with RNA-seq- or microarray-built models are shown as violin plots. (PDF) [file pcbi.1005921.s010.pdf]

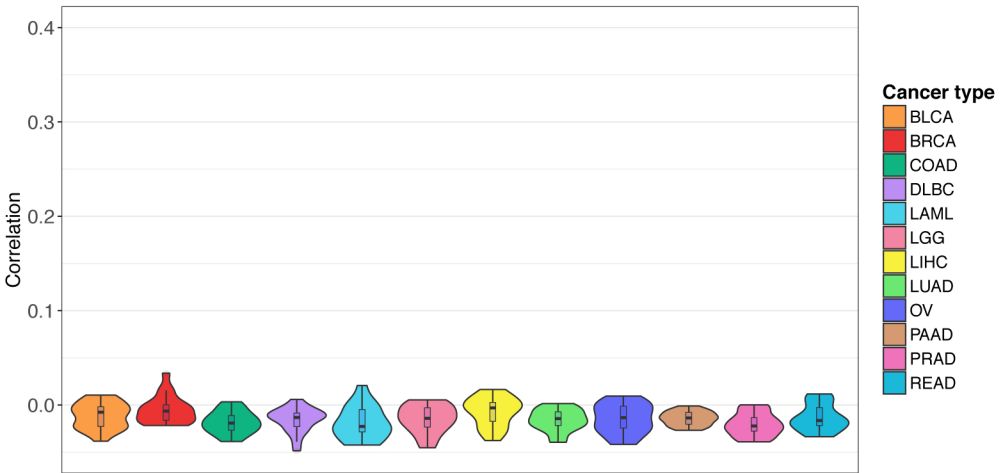

Supplement: S11 Fig — CNV absolute segment mean scores were computed for each as explained in Materials and Methods section. Model prediction absolute error for each gene are given by our predictive model using nucleotide and dinuclotide percentages computed in all the regulatory regions. Models were fitted on 16,294 genes for each of the 234 on 241 samples having CNV TCGA data available. The median correlation for the 234 samples is -0.014. (PDF) [file pcbi.1005921.s011.pdf]

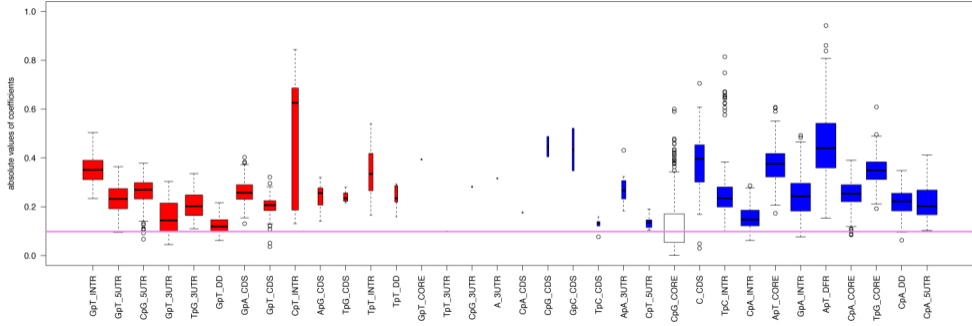

Supplement: S12 Fig — A linear regression model was built, for each sample, on standardized stable variables only. The boxplots show absolute values of the corresponding coefficients in all samples for each variable considered. Color code as in Fig 5. CpG in the core promoter is highlighted in white. Purple line represents the median of CpG_CORE coefficients. (PDF) [file pcbi.1005921.s012.pdf]

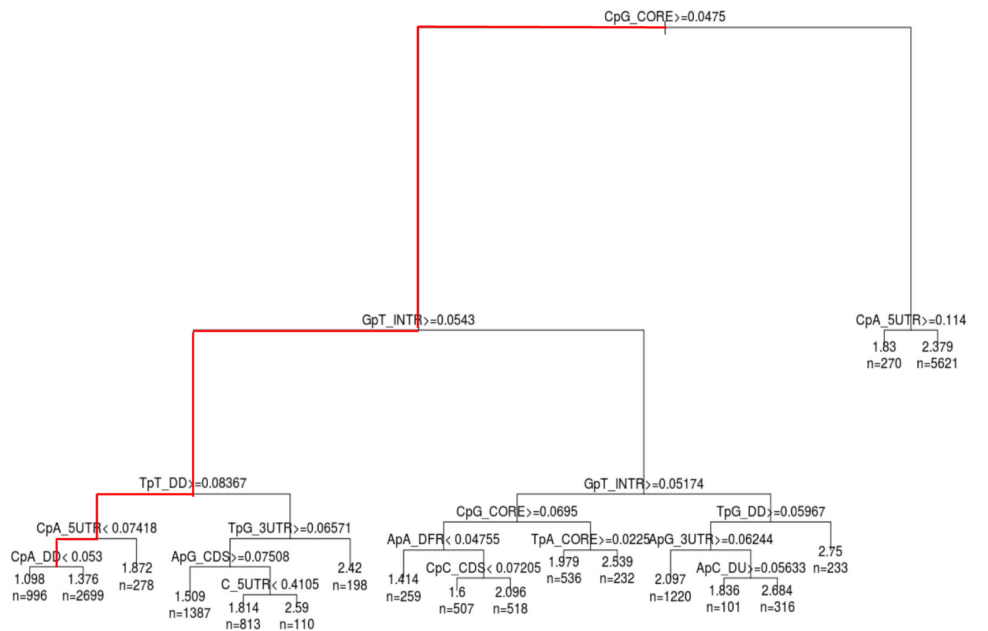

A

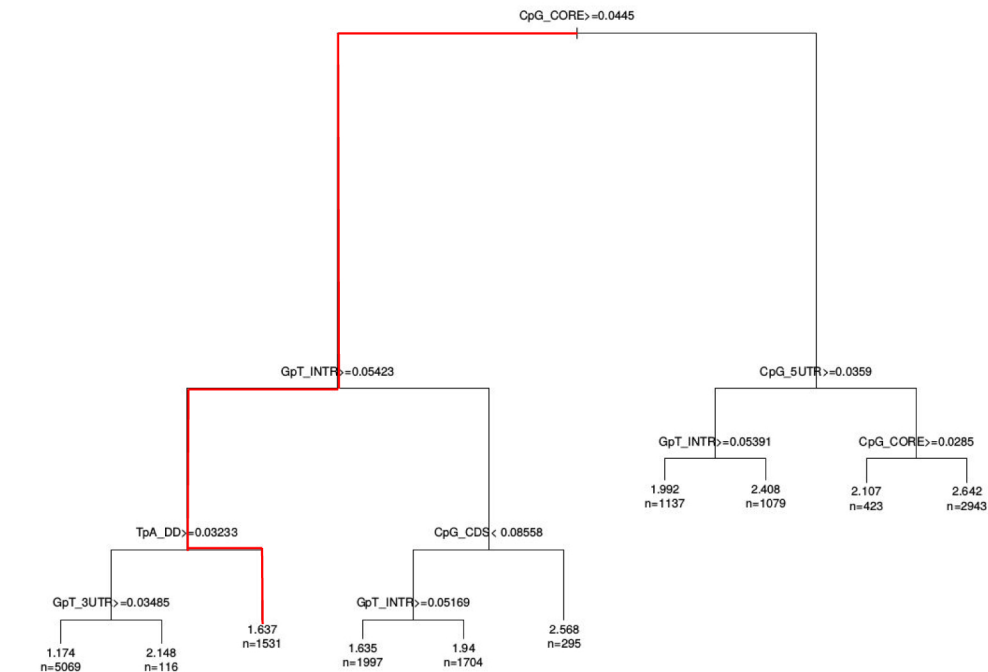

B

Supplement: S13 Fig — A: Regression tree leading to a group of genes well predicted in all samples. This tree has been learned on the sample TCGA.FC.A5OB.01A.11R.A29R.07_PRAD using all nucleotide composition in all regions. The red path defines a group of 996 genes which has low Lasso error in all samples and cancer types. This group was used for functional annotation (S4 Table). B: Regression tree leading to a group of genes well predicted in LGG and PPAD samples. This tree has been learned on the sample TCGA.IB.7646.01A.11R.2156.07_PAAD using all nucleotide composition in all regions. The red path defines a group of 1,531 genes which has low Lasso error in LGG and PAAD samples but high error in LAML, LIHC and DLBC samples. This group was used for functional annotation (S5 Table). (PDF) [file pcbi.1005921.s013.pdf]
