# Supplementary material for: Probing instructions for expression regulation in gene nucleotide compositions
Source: PLoS Comput Biol. 2018 Jan 2;14(1):e1005921. doi: 10.1371/journal.pcbi.1005921 (PMC5766238; doi:10.1371/journal.pcbi.1005921)
Supplement: S8 Fig — The procedure is identical to that described in Fig 4 but models were built on isoform-specific variables and correlations were computed between observed and predicted isoform expression, not gene expression. (PDF) [file pcbi.1005921.s008.pdf]

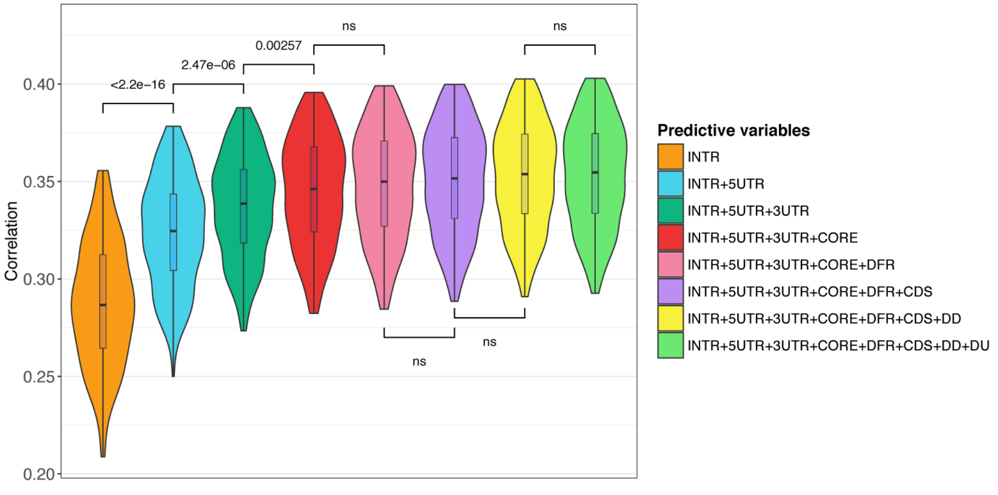

ns
ns
0.00257
2.47e−06
<2.2e−16
0.40
0.35
0.30
0.25
0.20
Correlation
ns
ns
Predictive variables
INTR
INTR+5UTR
INTR+5UTR+3UTR
INTR+5UTR+3UTR+CORE
INTR+5UTR+3UTR+CORE+DFR
INTR+5UTR+3UTR+CORE+DFR+CDS
INTR+5UTR+3UTR+CORE+DFR+CDS+DD
INTR+5UTR+3UTR+CORE+DFR+CDS+DD+DU
