# Supplementary material for: Probing instructions for expression regulation in gene nucleotide compositions
Source: PLoS Comput Biol. 2018 Jan 2;14(1):e1005921. doi: 10.1371/journal.pcbi.1005921 (PMC5766238; doi:10.1371/journal.pcbi.1005921)
Supplement: S1 Table — Each model is fitted for each tumor, using all the variables over all regions (160 variables among 8 regulatory regions). First and second columns are median correlation and mean square error over all the tumors. The third column represents mean computing time per tumor (in minutes) on a standard laptop. (PDF) [file pcbi.1005921.s014.pdf]

Method	Median correlation	Median error	Mean time
Linear model (with Lasso)	0.578	6.50	0.13
Random Forest	0.592	6.293	5.033
Regression trees	0.45	7.456	0.172
