# Supplementary material for: Probing instructions for expression regulation in gene nucleotide compositions
Source: PLoS Comput Biol. 2018 Jan 2;14(1):e1005921. doi: 10.1371/journal.pcbi.1005921 (PMC5766238; doi:10.1371/journal.pcbi.1005921)
Supplement: S3 Table — The procedure is identical to that described in S2 Table but models were built on isoform-specific variables and correlations were computed between observed and predicted isoform expression, not gene expression. (PDF) [file pcbi.1005921.s016.pdf]

| | INTR | 5UTR | 3UTR | CORE | DFR | CDS | DD | DU |
|---|---|---|---|---|---|---|---|---|
| STEP 1 | 0.2866 | 0.2462 | 0.1879 | 0.2417 | 0.1577 | 0.1392 | 0.1757 | 0.1358 |
| STEP 2 | | 0.3246 | 0.3080 | 0.3182 | 0.2918 | 0.2959 | 0.2931 | 0.2898 |
| STEP 3 | | | 0.3387 | 0.3348 | 0.3275 | 0.3286 | 0.3276 | 0.3248 |
| STEP 4 | | | | 0.3462 | 0.3416 | 0.3429 | 0.3416 | 0.3396 |
| STEP 5 | | | | | 0.3499 | 0.3487 | 0.3487 | 0.3458 |
| STEP 6 | | | | | | 0.3516 | 0.3500 | 0.3478 |
| STEP 7 | | | | | | | 0.3538 | 0.3515 |
| STEP 8 | | | | | | | | 0.3547 |
