# Supplementary material for: Probing instructions for expression regulation in gene nucleotide compositions
Source: PLoS Comput Biol. 2018 Jan 2;14(1):e1005921. doi: 10.1371/journal.pcbi.1005921 (PMC5766238; doi:10.1371/journal.pcbi.1005921)
Supplement: S4 Table — The group of 996 genes is obtained by fitting a regression tree on the sample TCGA.FC.A5OB.01A.11R.A29R.07_PRAD using all the nucleotide composition in all regions. These genes are well predicted (mean error < 1st quartile) for all samples of different type cancers. This group of genes was further annotated using the DAVID functional annotation tool. Only the top 5 biological processes indicated by DAVID is shown. The GO term yielded by this analysis corresponded to general and widespread biological processes indicating that these genes likely corresponded to housekeeping genes. (PDF) [file pcbi.1005921.s017.pdf]

Gene ontology term	Count	Benjamini corrected P-value
Cellular macromolecule metabolic process	612	1.8E-23
Cellular metabolic process	681	1.2E-16
Cellular protein metabolic process	390	2.8E-16
Macromolecule metabolic process	624	4.0E-16
Nucleic acid metabolic process	404	4.0E-16
