# Supplementary material for: Probing instructions for expression regulation in gene nucleotide compositions
Source: PLoS Comput Biol. 2018 Jan 2;14(1):e1005921. doi: 10.1371/journal.pcbi.1005921 (PMC5766238; doi:10.1371/journal.pcbi.1005921)
Supplement: S5 Table — The group of 1,531 genes is obtained by fitting a regression tree on the sample TCGA.IB.7646.01A.11R.2156.07_PAAD using all the nucleotide composition in all regions. These genes are well predicted (mean error < 1st quartile) for all LGG and PAAD samples but not that of LAML, DBLC and LIHC. This group of genes was further annotated using the DAVID functional annotation tool. Only the top 5 biological processes indicated by DAVID is shown. The GO term “Nervous system development” indicates that these genes can be involved in specific biological processes. (PDF) [file pcbi.1005921.s018.pdf]

Gene ontology term	Count	Benjamini corrected P-value
Positive regulation of cellular process	528	7.0E-14
Nervous system development	284	1.3E-13
Positive regulation of macromolecule metabolic process	346	3.5E-12
Positive regulation of biological process	565	8.1E-12
Neurogenesis	200	5.9E-11
