# Supplementary material for: Probing instructions for expression regulation in gene nucleotide compositions
Source: PLoS Comput Biol. 2018 Jan 2;14(1):e1005921. doi: 10.1371/journal.pcbi.1005921 (PMC5766238; doi:10.1371/journal.pcbi.1005921)
Supplement: S6 Table — We computed the density of cis-eQTL per regulatory region by dividing the sum of cis-eQTLs intersecting with the region considered for all genes by the sum of the lengths of the same regulatory region of all genes. see Material and methods for details. (PDF) [file pcbi.1005921.s019.pdf]

	CORE	5UTR	CDS	3UTR	INTR	DFR
**eQTL density**	$5.29 * 10^{-4}$	$5.54 * 10^{-4}$	$1.28 * 10^{-4}$	$1.78 *^{-4}$	$7.03 * 10^{-5}$	$1.49 * 10^{-4}$
