# Supplementary material for: Probing instructions for expression regulation in gene nucleotide compositions
Source: PLoS Comput Biol. 2018 Jan 2;14(1):e1005921. doi: 10.1371/journal.pcbi.1005921 (PMC5766238; doi:10.1371/journal.pcbi.1005921)
Supplement: S7 Table — We computed the density of SNPs per regulatory region by dividing the sum of SNPs intersecting with the region considered for all genes by the sum of the lengths of the same regulatory region of all genes. We only considered SNPs detected on chromosomes 1, 2 and 19. see Material and methods for details. (PDF) [file pcbi.1005921.s020.pdf]

SNP density	CORE	INTR
**chr1**	0.03937541	0.02680815
**chr19**	0.05870189	0.04408599
**chr2**	0.06016752	0.04202322
