# Supplementary material for: Probing instructions for expression regulation in gene nucleotide compositions
Source: PLoS Comput Biol. 2018 Jan 2;14(1):e1005921. doi: 10.1371/journal.pcbi.1005921 (PMC5766238; doi:10.1371/journal.pcbi.1005921)
Supplement: S8 Table — We computed the density of enhancers per regulatory region by dividing the total length of the intersection between the enhancers and the region considered for all genes by the sum of the lengths of the same regulatory region of all genes. see Material and methods for details. (PDF) [file pcbi.1005921.s021.pdf]

	5UTR	CDS	3UTR	INTR	DFR
**enh density**	$8.27 * 10^{-4}$	$2.44 * 10^{-5}$	$6.41 * 10^{-4}$	$5.14 *^{-3}$	$4.08 * 10^{-3}$
